# Evolutionary and Ecological Drivers of Gut Microbiota in Wild Rodent Species from the Yucatán Peninsula

**DOI:** 10.1007/s00248-025-02603-3

**Published:** 2025-09-30

**Authors:** Gabriela Borja-Martínez, Arit de León-Lorenzana, Alfredo Yanez-Montalvo, Giovani Hernández-Canchola, Luisa I. Falcón, Ella Vázquez-Domínguez

**Affiliations:** 1 Laboratorio de Genética y Ecología, Departamento de Ecología de La Biodiversidad, Instituto de Ecología, Universidad Nacional Autónoma de México, 04510 Ciudad de México, Mexico; 2 Posgrado en Ciencias Biológicas, Universidad Nacional Autónoma de México, 04510 Ciudad de México, Mexico; 3 Laboratorio de Ecología Bacteriana, Instituto de Ecología, Unidad Mérida, Universidad Nacional Autónoma de México, 97357 Ucú, Yucatán Mexico; 4 Universidad Intercultural Maya de Quintana Roo-CONAHCYT, Carretera Muna-Felipe Carrillo Puerto Km 137, La Presumida, José María Morelos, 77870 Quintana Roo, Mexico; 5 Facultad de Ciencias, Fraccionamiento Playitas, Universidad Autónoma de Baja California, Carretera Transpeninsular 3917, 22860 Ensenada Baja California, Mexico; 6 Museo de Zoología Alfonso L. Herrera, Departamento de Biología Evolutiva, Facultad de Ciencias, Universidad Nacional Autónoma de México, 04510 Ciudad de México, Mexico

**Keywords:** Coevolution, Mexico, Microbial diversity, Phylosymbiosis, Sympatric host species

## Abstract

The host-microbiome association is considered a coevolutionary process, in which the microbiome provides important functions for host development, physiology and health. However, the ecological and evolutionary forces shaping the diversity and structure of the bacterial communities that form the microbiome are still being elucidated. We assessed the composition of gut microbiota in six rodent species from three geographic regions across the Yucatán peninsula, Mexico. We evaluated the contribution of host species identity, phylogenetic relationships, and geography to the rodents’ gut microbiota, using 16S rRNA V4 sequences. We performed a comprehensive set of analytical approaches, including Hill numbers, machine learning, and phylogenetic comparative frameworks. Our results show that phylosymbiosis is one of the main mechanisms driving microbiota dissimilitude across species and specific microbiota diversity traits. Additionally, the microbial pool in each region was geographically differentiated, shaped by the rodent community ensemble, while ecological filtering rendered a microbial pool characteristic of each species. The environment also played a significant role for some species like *Heteromys gaumeri*, while dietary habits showed a stronger signal for *Oryzomys couesi*. Rodents with more specialized habits like *Ototylomys phyllotis* (semi-arboreal, folivorous) had higher bacterial diversity. The abundance of eight bacterial families determined key differences of the gut microbiota which, in addition to phylogeny and geography, are associated with distinct diet and metabolic functions among rodents. Distinct metabolic functions were related, among others, to toxins metabolism and digestion of complex food components. Overall findings show that both evolutionary and ecological drivers influence these rodents gut microbial structure and composition.

**Supplementary Information:**

The online version contains supplementary material available at 10.1007/s00248-025-02603-3.

## Introduction

The close relationship between the microbiome and the host is considered a coevolutionary process [1, 2] in which microorganisms can, for instance, confer the ability to develop new metabolic functions and colonize new environments [3, 4]. Multiple ecological and evolutionary processes acting at different spatial and temporal scales play a fundamental role in shaping the diversity and structure of the gut microbiota [5, 6]. Phylogeny, biogeography, and speciation are key evolutionary aspects [7, 8]. Ecological factors include specific characteristics of the species, such as diet, sex, behavior, and developmental stage, as well as social networks and biotic interactions [9, 10]. Notably, how these processes and factors interact in shaping the diversity and structure of microbial communities across diverse animal hosts is not fully understood. Geography and environmental heterogeneity shape the spatial variation of the gut microbiota, e.g., geographic barriers limit dispersal [5, 11], and, because the environment and food items vary throughout the space, the pool of microbiota available to colonize the host differs accordingly. Therefore, similar microbial communities among sympatric populations or species are expected [2, 8, 12].

Phylosymbiosis refers to the eco-evolutionary pattern in which closely related host species often tend to have a more similar microbiota, and where microbiota dissimilitude between host species is associated with host divergence time [13]. Namely, the overall microbial communities or a subset of multiple microbial lineages recapitulate the host phylogeny [14]. Phylosymbiosis implies reciprocal adaptation between microbiota and host, which can result from stochastic evolution and ecological drift due to changes in the host geographic range, host population density, and host microbial population size, among others [13–15]. It can also result from direct host regulation of microbial lineages by host genetics or host environment, or be associated with factors like diet (different bacterial communities from distinct food items) [14, 16, 17]. Some animal groups exhibit phylosymbiosis more consistently than others, like salamanders [18], birds [19], and mammals [17, 19–22]. Nonetheless, rodent patterns have been shown to differ and exhibit context-specific responses. Some studies with mice found that diet [23], geography [24, 25], environment [7, 26], host genetics [25], or phylogeny [20] are the main factors shaping microbial diversity. Hence, a more comprehensive understanding of the interaction of different factors that govern host-microbe interactions in rodents is needed.

We aimed to assess the composition of gut microbiota in six sympatric rodent species from three geographic regions across the Yucatán peninsula. We evaluated the relative contribution of host species identity, phylogenetic relationships, habits, and geographic region to the diversity and structure of the rodents’ gut microbiota. Under our sampling scheme we propose two possible, non-exclusive, evolutionary (host species identity and phylogeny) and ecological (geography and host habits) scenarios: (i) if the host species identity is the primary driver of gut microbial structure and diversity, we predict that each host species would have an overall distinct bacterial community, despite being in the same environment. We also ask whether microbial communities recapitulate the host phylogeny, indicating phylosymbiosis; (ii) considering that the study regions exhibit distinct environmental features and availability of food items, we propose that host overall gut microbial structure and diversity are likely determined by geographic region; additionally, where sympatric host species share the same environment within regions, they will share specific microbial taxa.

## Methods

### Study Site and Sampling

**Fig. 1:**
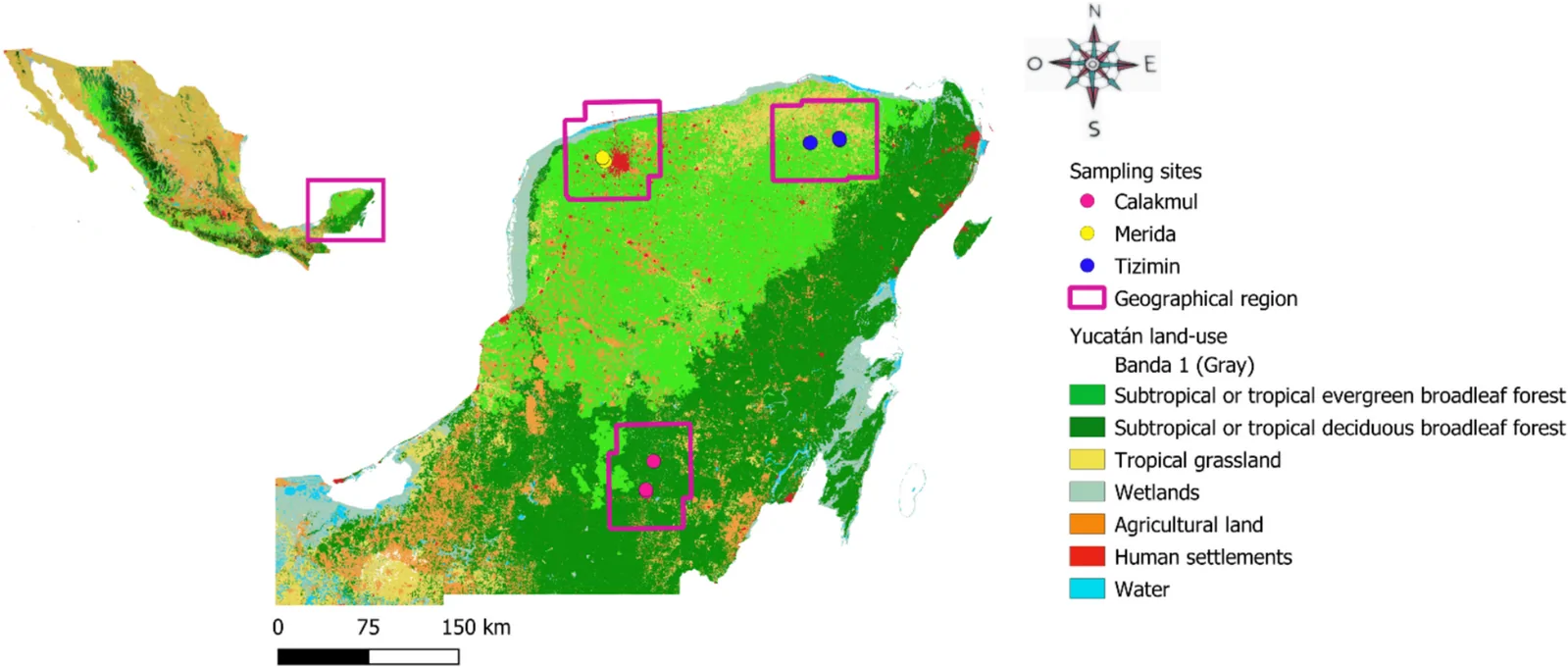
Map of geographic regions and sampling sites. The three study regions are depicted with pink-lined polygons: Mérida (urban), Tizimín (rural), and Calakmul (natural); sampling sites are indicated with yellow, blue, and red circles, respectively. The classification of main vegetation types and land uses are shown, based on public domain information [29], and we created the map using QGIS 3.26.2 software

The Yucatán peninsula in Mexico (145,000 km^2^) has a distinctive geology and hydrogeology, characterized by little soil cover and limited nutrient availability. Although rainfall is abundant, water is scarce due to the absence of superficial streams. Average annual rainfall increases from northwest to southeast and, following this gradient, vegetation changes from dry forest to semi-evergreen forest to rainforest [27]. It is a biogeographic province, harboring endemic flora and fauna, including 8% of Mexican small rodent species (15 species) [28]. We targeted sampling in three geographic regions (Fig. 1): i) Mérida (921,771 inhabitants) is the political, cultural and economic hub of the region; ii) Tizimín ('the tapir’s place' in Maya; 52,600 inhabitants) is the primary livestock producer in the peninsula; iii) Calakmul ('two adjacent mounts'), is a natural and cultural World Heritage site recognized by UNESCO and a Biosphere Reserve that comprises the largest tropical forest (dry, tall and medium-height subperennial rainforest) in Mexico [29]. Thus, these regions encompass distinct environments and food items availability for rodents.

The rodent community was live-trapped in three sites within each region, for two nights per site, using 120 Sherman traps (baited with a mixture of oats, vanilla extract, and peanut butter) spaced 10 m apart along randomly set transects. Sampling was performed in the dry season in November 2021 (Mérida), March 2022 (Tizimín), and May 2022 (Calakmul). We replaced each trap with a clean one after rodent capture, following strict protocols to prevent cross-contamination. Each individual was identified to species, sexed, and weighed, standard morphological measures were taken, and fresh fecal material was collected directly from the rectum, at the moment of defecation, in Eppendorf tubes and preserved in liquid nitrogen (Supplementary Table S1). We trapped six rodent species: the Gaumer's spiny pocket mouse (*Heteromys gaumeri*), the Coues's rice rat (*Oryzomys couesi*), which is a semi-aquatic rodent, the big-eared climbing rat (*Ototylomys phyllotis*) an arboreal species, the white-footed mouse (*Peromyscus leucopus*), the Yucatán deer mice (*Peromyscus yucatanicus*), and the Toltec cotton rat (*Sigmodon toltecus*), the three with terrestrial habits (Table 1, Table S1). One sample, from the fulvous pygmy rice rat (*Oligoryzomys fulvescens*), was sequenced, and its overall gut microbiota composition is described since it is the first data available for this species (Supplementary Fig. S1), but it was not used for analyses. Animals were released at the sampling site, assuring individual well-being (see Extended methods Supplementary material for more detailed descriptions).

**Table 1:** Sampled rodent species at the three regions studied (Calakmul, Tizimín, Mérida) in the Yucatán peninsula, Mexico. Sample size (*N*), distribution within regions, sex, and age are indicated

Species	*N*	Geographic region		Sex		Age	
Heteromys gaumeri	7	Calakmul	2	Female	3	Juvenile	2
		Tizimín	3	Male	4	Adult	5
		Mérida	2				
Oryzomys couesi	3	Calakmul	3	Female	2	Juvenile	1
				Male	1	Adult	2
Ototylomys phyllotis	12	Calakmul	7	Female	9	Juvenile	2
		Tizimín	5	Male	3	Adult	10
Peromyscus leucopus	4	Tizimín	1	Female	1	Juvenile	3
		Mérida	3	Male	3	Adult	1
Peromyscus yucatanicus	18	Mérida	17	Female	10	Juvenile	7
		Tizimín	1				
				Male	8	Adult	11
Sigmodon toltecus	17	Calakmul	1	Female	9	Juvenile	2
		Tizimín	15	Male	8	Adult	15
		Mérida	1				

### DNA Extraction and Sequencing

DNA was extracted from fecal samples using the QIAamp PowerFecal Pro DNA Kit (Qiagen) following the manufacturer´s instructions. Total DNA per sample was quantified in Qubit and all samples were diluted to 20 ng/µl. We amplified the V4 variable region of the 16S rDNA locus using primers 515F/806R [30] in triplicate per sample. Negative controls included the different buffers used for the DNA extraction and amplification, which did not generate any PCR product; thus, they were not sequenced. The libraries were sequenced on Illumina MiSeq in the Yale Center for Genome Analysis (YCGA) (see Extended methods).

### Bioinformatics Processing

Reads were processed using a semi-automated pipeline in QIIME2. Reads were demultiplexed and paired; low-quality reads (scores < 30), singletons, and chimeras were discarded and truncated at 114 bp. We assigned reads to ASVs (Amplicon Sequence Variants) and taxonomy was resolved using the SILVA 138 database [31]. We performed de novo alignment by similarity with MAFFT [32], and a phylogenetic tree was built using FastTree [33] with the approximate likelihood method. ASVs assigned to mitochondria or chloroplast were removed from the feature table. All following analyses were performed using different packages in the R environment v.4.2.2 [34]. We estimated species accumulation curves by sample in phyloseq 1.42 [35], and data were rarefied to 5,000 reads per sample to correct for different read depths. We used the rarefied dataset for alpha and beta diversity estimates, microbial composition, and enrichment. A non-rarefied dataset, in which samples with less than 2,000 reads were eliminated, was used for phylogenetic signal analyses.

### Microbial Abundance, Alpha and Beta Diversity

To characterize the gut microbiota, we assessed abundance differences of microbial taxa at the family level among rodent host species with a linear discriminant effect size analysis (lefse) using microbiomeMarker v.1.4 [36]. We further identified the core gut microbiota and the shared taxa at the family level based on their relative abundances and on their 95% and 75% prevalence, with microbiome v.1.2 [37]. We also evaluated the unique and shared taxa at the genus level with microViz [38] and the DrawVenn tool available online (https://bioinformatics.psb.ugent.be/webtools/Venn/).

We quantified alpha diversity and phylodiversity across all taxonomic levels using Hill numbers with Hilldiv v1.5.1 [39], a method that measures diversity in units of effective ASVs (or effective lineages for phylodiversity) and includes three metrics: q0 that is the equivalent to species richness, q1 is the number of common taxa (the exponential of the Shannon entropy index), and q2 the number of dominant taxa (similar to Simpson’s reciprocal index) [39, 40]. The phylodiversity Hill numbers incorporate branching patterns, relative branch length, and branch abundance [39], thus representing measures of richness, common taxa, and dominant taxa (equivalent to: q0 = Faith´s PD, q1 = Allen’s Hp, and q2 = Rao’s Q). To estimate differences among host species and geographic regions, the appropriate Wilcoxon or Kruskal–Wallis tests were applied with Hilldiv.

To corroborate if the identity of the host species is the main structuring driver, we used a machine learning approach. Classification algorithms analyze, identify, classify, and predict a set of data based on their similarity. The abundance of bacterial family data was randomly partitioned into training and validation sets, containing 70% and 30% of the samples respectively, and bacterial families were used as predictors. We eliminated families that were correlated and, because of our unbalanced sampling, for the analysis based on host species, we eliminated the *O. couesi* samples and grouped the *P. leucopus* and *P. yucatanicus* samples, given their close phylogenetic relationship. We kept all the data for the analysis based on geographic region.

We implemented Support Vector Machines (SVM) and Random Forest (RF) algorithms. Machine learning helps in understanding underlying data structure and community-level patterns that are not captured by univariate methods like lefse. SVM seeks to find a straight line or hyperplane that separates the dataset while maximizing the minimum distance between points [41]. RF is an ensemble learning method composed of a set of decision trees that are trained independently [42]. Decision trees assess the best split to create subsets of data, in which each generated tree uses a random subset of features to avoid correlation between trees. The greater the number of decision trees, the less the classifier will overfit the model, because the average of uncorrelated trees reduces both the overall variance and the prediction error. We ran the two algorithms in python with sklearn.svm and sklearn.ensemble, respectively [43]. The best set of hyperparameters for each model was chosen with sklearn.model_selection (SVC = gamma = 0.001, C = 10, max_iter = 2500). We evaluated the performance of each algorithm based on the confusion matrix and calculated the importance of variables using sklearn.inspection.

To assess microbial community structure, we estimated Beta diversity with phyloseq, using the weighted (Wunifrac) and unweighted Unifrac (Unifrac) phylogenetic distance dissimilarities; unweighted considers taxa presence/absence while weighted considers their abundances [44]. We also estimated the Bray–Curtis index, which does not consider phylogeny [45]. We evaluated differences (a) within each host species by sex, age, and geographic region; (b) among host species and geographic regions, with a pairwise permutational analysis of variance (permanova) using the adonis function in vegan v.2.6.6 [46], and the dispersion or distance of centroid with betadisper. We implemented a model selection framework using the Akaike Information Criterion corrected for small sample sizes (AICc) for Permanova with AICcPermanova v.0.0.2 [47], to determine which variable best explained the structure of the microbiota. We used the Omega-squared values in MicEco [48] to estimate the Permanova effect size considering small sample sizes. We also did principal coordinate analysis (PCoA) and constructed a UPGMA tree to assess microbial community structure among host species and geographic regions.

### Phylogenetic Relationships

To evaluate the phylogenetic signal, we first obtained a phylogenetic tree for the rodent species studied. We downloaded cytochrome *b* sequences available in GenBank: *H. gaumeri* (Accession number: GU646999), *P. leucopus* (DQ973104), *O. phyllotis* (DQ179814), *S. toltecus* (EU073182), *O. fulvescens* (EU258547), and *O. couesi* (DQ185386). No sequence was available for *P. yucatanicus*; thus, we amplified, sequenced, and aligned one sample (GenBank: PQ742163), following [49]. We selected the best model and partition scheme, divided by codon positions, with PartitionFinder2 [50]. We built an ultrametric tree with BEAST 1.10.4 [51]; convergence of runs was evaluated with Tracer 1.7 [52], results were combined with LogCombiner, and a maximum credibility tree was built with TreeAnnotator (see Extended methods).

Microbial communities are assumed to be phenotypic traits of the host with continuous variation [53]. We evaluated the statistical independence of traits among host species, considering their phylogenetic relationships obtained in our phylogenetic tree [54]. Specifically, we tested whether alpha and phylodiversity (q0, q1, q2) and beta dispersion (distance to the centroid), calculated as the Wunifrac and Bray–Curtis distances from each sample to the sample-set centroid across all bacterial taxonomic levels, showed a phylogenetic signal. Based on the phylogenetic comparative framework, we applied Blomberg’s K and Pagel’s λ tests with phytools [55]. Both tests compare if trait similarity among species is greater than expected following a random-walk model (Brownian motion) along the branches of a phylogeny [14, 55]; and quantify the degree to which a trait is explained by the structure of a phylogeny, where K > 1 means that closely related species are more similar than predicted by the Brownian model. Additionally, we built a phenetic tree for each microbial trait (q0, q1, q2) and taxonomic level (Class, Order, Family, ASVs) with phytools, based on the rodent species' similarity. Finally, to evaluate the phylogenetic signal of the beta diversity trait, we performed a correlative Mantel test between host phylogenetic distances and bacterial community dissimilarity using vegan.

## Results

We trapped six rodent species from two families, Heteromyidae (*H. gaumeri*) and Cricetidae (O. *couesi*, *O. phyllotis, P. leucopus, P. yucatanicus, S. toltecus*)*.* A total of 72 rodent fecal samples were collected, from which 16S rRNA amplicons were successfully obtained for 61 (Calakmul = 13, Tizimín = 25, Mérida = 23). Sequencing yielded a total of 9,698,211 reads (mean = 146,942); 78% were retained per sample after quality filtering, resulting in 7,011,727 reads (min = 5, mean = 106,238). Both the rarefied and non-rarefied datasets included the six rodent species and 61 samples, the former comprising 5878 unique ASVs distributed across 15 phyla, 28 classes, 65 orders, and 162 families. The non-rarefied dataset used for the phylogenetic signal analysis contained 9357 ASVs distributed in 15 phyla, 54 classes, 174 orders, and 327 families, irrespective of host species.

### Interspecific Gut Microbiota Composition

**Fig. 2:**
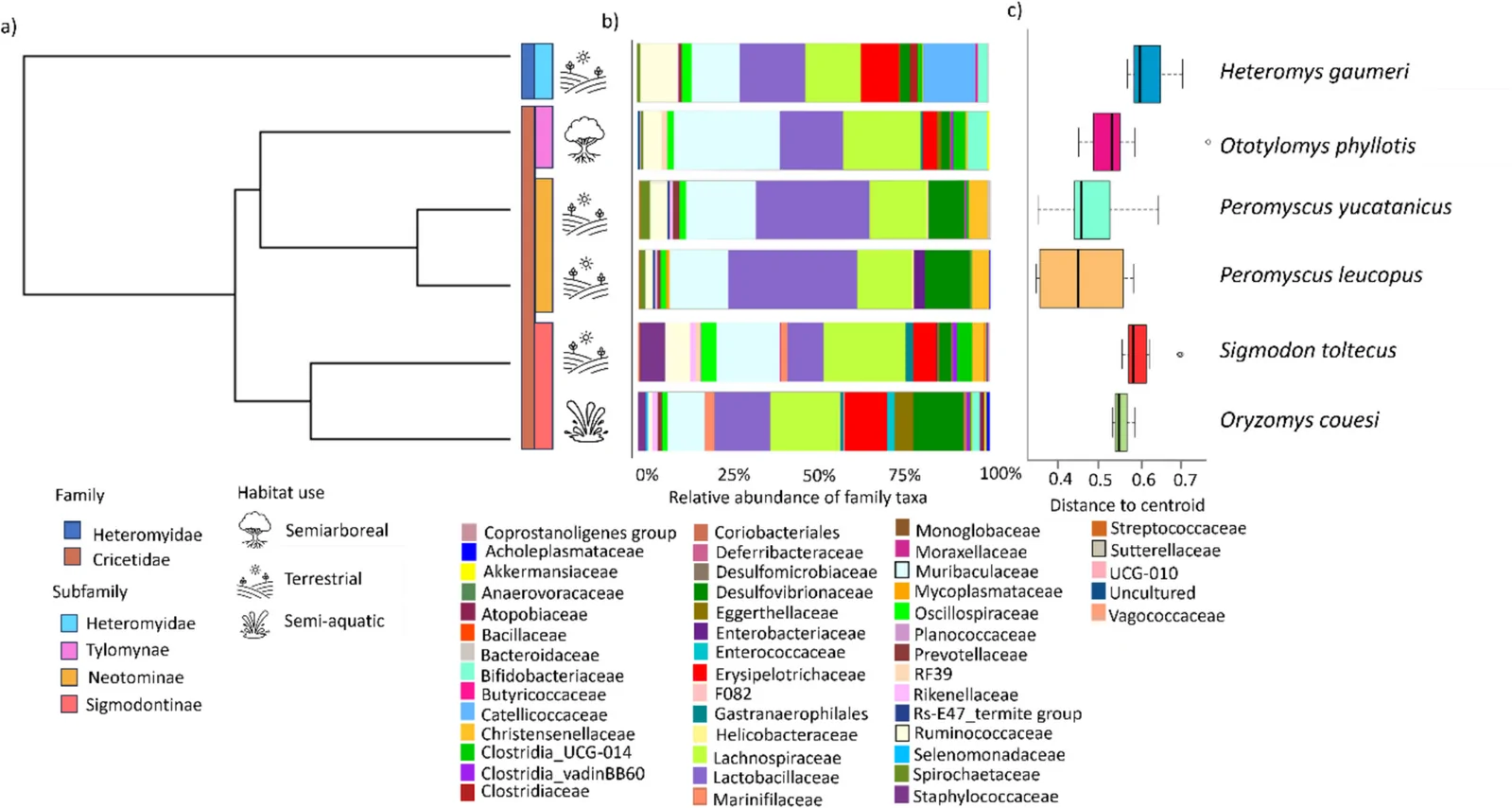
** a** Phylogeny of the rodent species studied based on cytochrome *b* sequences. Rodent taxonomic classification (Family and Subfamily) and habitat use (semiarboreal, terrestrial, semi-aquatic) of each species are indicated. **b**) Relative abundance of bacterial families (higher than 0.01; colored bars) of the gut microbiota of each host species. **c**) Boxplot showing bacterial beta dispersion (distance to the centroid) per host

**Fig. 3:**
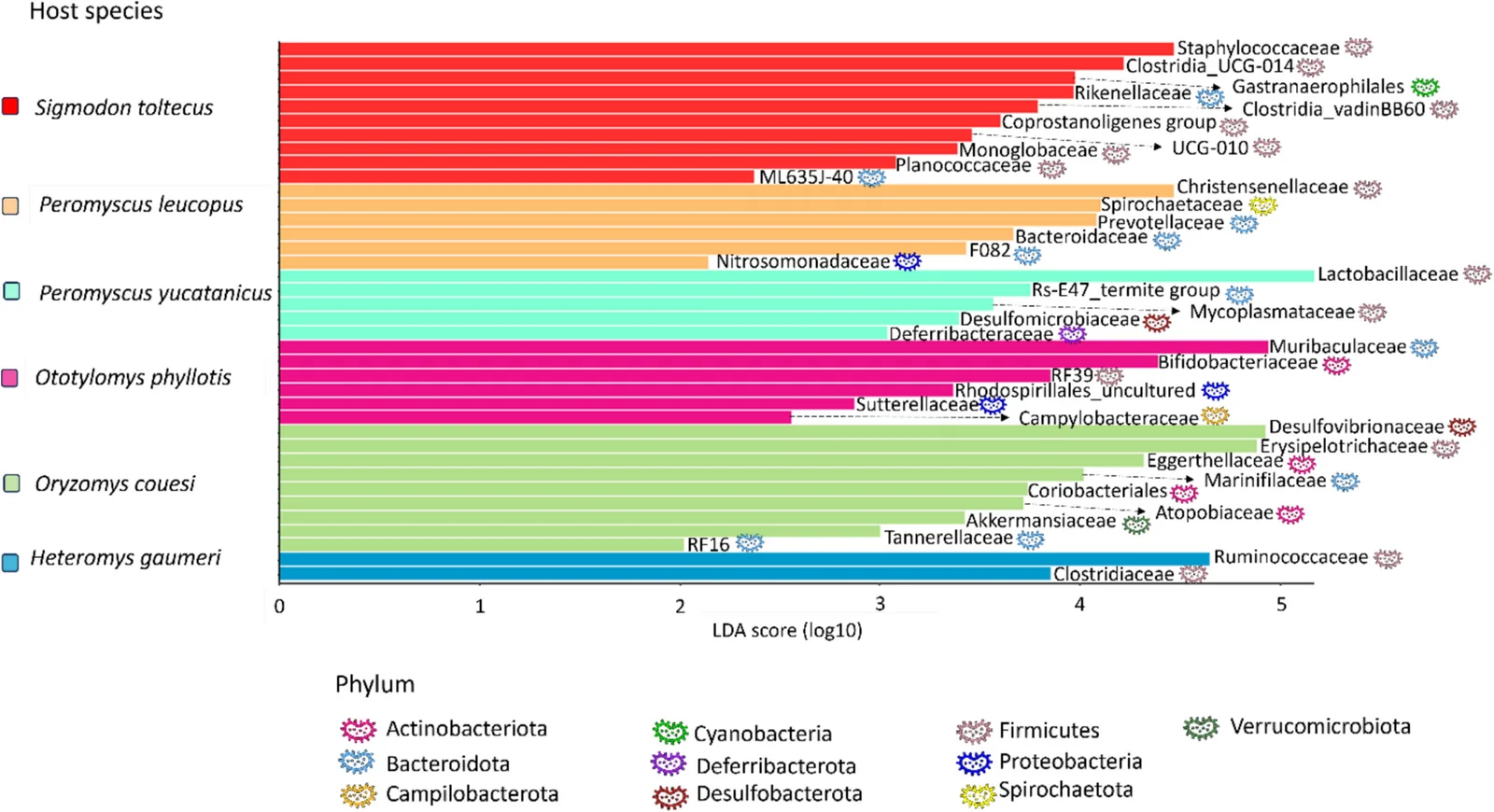
Gut microbiota differences among host species. Linear discriminant analysis effect size plots of the identified characteristic gut microbiome taxa at the family level and the phylum to which they belong of each host rodent species

Three bacterial phyla accounted for over 93% of the interspecific variation, distributed among Firmicutes (mean 64.6%), Bacteroidota (22.62%), and Desulfobacterota (6.2%) (Fig. S2); the rest of the microbial diversity was comprised by other 12 phyla, including Actinobacteriota, Spirochaetota, Verrucomicrobiota, Campilobacterota, and Proteobacteria. Within Firmicutes, class members of Bacilli (46.35%) and Clostridia (53.31%) were prevalent, while Bacteroidota was represented by a single class, Bacteroidia. Four families constituted the microbial core with 95% prevalence across host species: Lachnospiraceae, Lactobacillaceae, Muribaculaceae, and Ruminoccocaceae (Fig. 2). Six additional families were present in the microbial core based on 75% prevalence: Clostridia UCG-014, Christensenellaceae, Desulfovibrionaceae, Eggerthellaceae, Oscillospiraceae, and Rikenellaceae. The lefse analysis (Fig. 3) identified 38 bacterial families whose relative abundances explained the microbial differences among hosts. Specifically, Lactobacillaceae was highly abundant in *P. yucatanicus*, Christensenellaceae predominated in *P. leucopus*, Muribaculaceae in *O. phyllotis*, Ruminoccocaceae in *H*. *gaumeri*, Desulfovibrionaceae in *O. couesi*, and Staphylococcaceae in *S. toltecus*. Finally, all host species, except *P. leucopus*, showed unique taxa at genus level, where *S. toltecus* had the highest number (> 50 genera) (Table S2).

We used machine learning as an alternative approach for assessing differences between gut bacterial composition based on host species and geographic region. Both vector machine (SVM) and Random forest (RF) were able to categorize the individuals to their respective species group with high accuracy (RF 93–100%; SVM 74–80%) for all species (Table S3); only *H. gaumeri* individuals could not be classified correctly (performance 0–60%). RF results indicated that the main differences are given by eight families, in agreement with lefse results: Prevotellaceae, Lactobacillaceae, and Rs-E47_termite_group, Erysipelotrichaceae, Eggerthellaceae, and Marinifilaceae for the *Peromyscus* group; Bifidobacteriaceae for *O. phyllotis*; and Rikenellaceae for *S. toltecus* (Figs. 2,3). These findings corroborate that the gut bacterial composition differed significantly among host species. Regarding region (mean = 83%), Mérida was classified with high performance (96%), Tizimín average classification was > 80% and Calakmul had low performance (0–46%).

### Interspecific Gut Microbiota Diversity

The alpha diversity and phylodiversity profiles of the microbial community differed in function of the bacterial taxonomic category (ASVs, Family, Order; Table 2). Significant differences (*p* < 0.01) among rodent species were found for ASVs, where *O. phyllotis* and *S. toltecus* showed the highest richness (q0) and *P. leucopus* the lowest across all taxonomic levels (Fig. S3). The resulting slope between q1 and q2 in the diversity profile indicated that the number of rare or low-abundance species is greater for *O. phyllotis* and *S. toltecus*. No differences were observed for common (q1) and dominant (q2) taxa for any taxonomic category. We also found significant differences for alpha diversity (ASVs) by region for q0 and q1; specifically, Tizimín and Mérida showed highest and lowest richness, respectively (Fig. S4). Finally, phylodiversity at the family level showed different patterns among host species (Fig. S5).

**Table 2:** Alpha diversity and phylodiversity at three taxonomic levels (ASVs, Family, Order) by host species; q0: species richness, q1: number of common species, q2: number of dominant species

Species									
	ASVs			Family			Order		
	q0	q1	q2	q0	q1	q2	q0	q1	q2
*Heteromys gaumeri*	223	24.49	4.82	32.85	5.11	2.82	23.28	4.30	2.71
	113.6	6.57	2.07	19.78	2.93	1.78	15.10	2.67	1.75
*Ototylomys phyllotis*	480.5	86.12	16.6	33.33	7.08	4.15	23.41	6.32	4.03
	240.1	12.53	2.53	20.84	3.65	2.05	15.68	3.42	2.03
*Peromyscus yucatanicus*	242	43.65	11.0	32.40	7.00	4.24	20.06	5.22	3.70
	156.2	16.64	4.30	24.39	5.09	2.99	17.00	4.23	2.80
*Peromyscus leucopus*	183	40.45	11.4	29.25	6.03	3.53	18.75	4.98	3.34
	92.01	8.69	2.48	17.49	3.28	1.95	12.60	2.97	1.92
*Oryzomys couesi*	234	48.73	18.5	33.66	8.05	4.62	24.0	6.05	4.08
	110.0	9.35	2.61	20.19	3.82	2.10	15.72	3.31	2.04
*Sigmodon toltecus*	400.5	68.06	9.01	35.73	7.35	4.04	23.26	5.55	3.56
	205.8	11.59	2.38	21.37	3.65	2.01	15.07	3.14	1.94
*p*-value	**0.001**	**0.031**	0.16	0.513	0.41	0.66	0.074	0.15	0.83
	**0.001**	**0.039**	0.53	0.305	0.55	0.86	0.089	0.21	0.88

For each species, the number above and below represents alpha diversity and phylodiversity

**Fig. 4:**
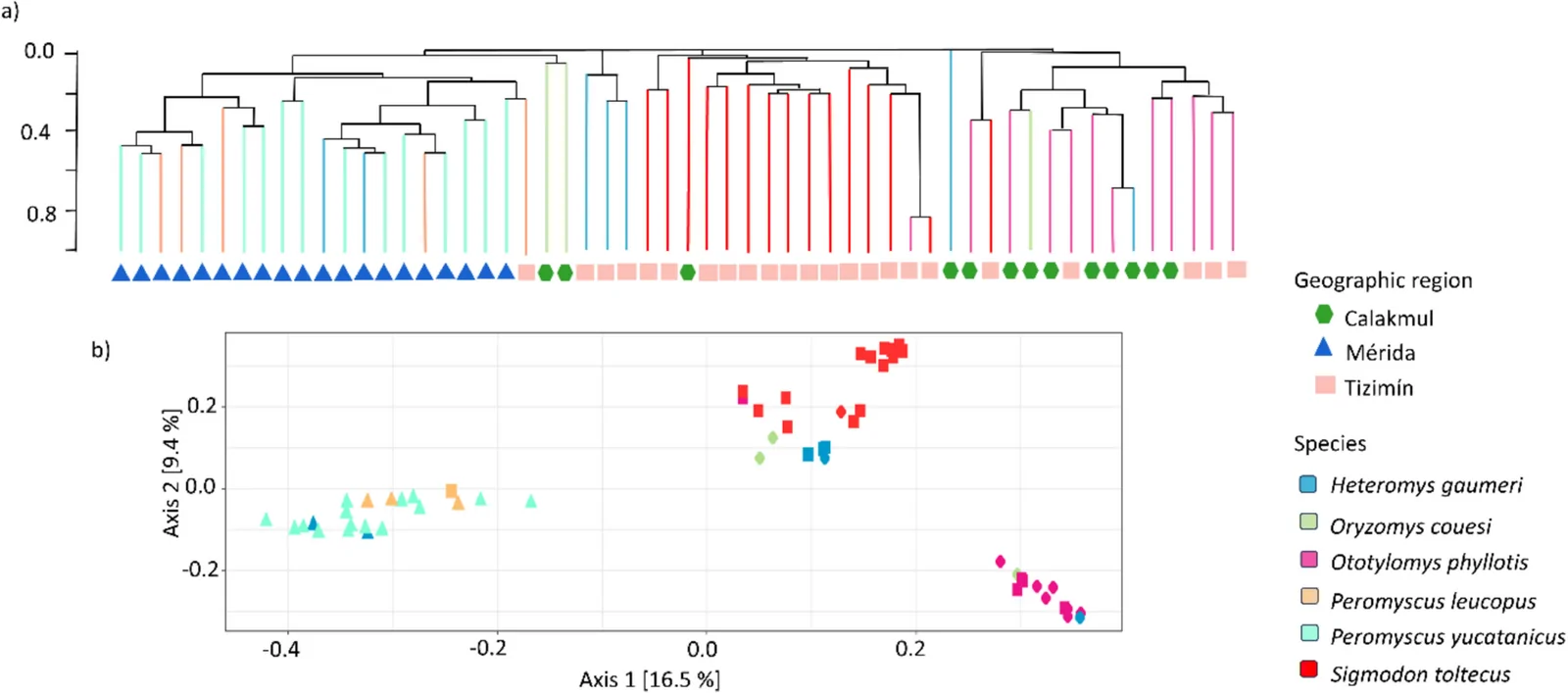
Bacterial diversity for the six rodent host species. **a** UPGMA line colors represent the different host species and geometric figures below the lines depict the geographic regions. **b** principal coordinate analysis (PCoA) of the dissimilarities among host species based on Bray–Curtis index. Colors represent the different host species and geometric figures the geographic region

The Permanova results for gut microbiota beta diversity showed that region (Bray–Curtis index and weighted Unifrac distance dissimilarities) and host species (unweighted Unifrac) were the main factors explaining community structure (Fig. 4; Table 3). Likewise, at the species level, unweighted Unifrac distances showed significant differences between *P. yucatanicus* and *O. phyllotis* and between *P. yucatanicus* and *S. toltecus*. No gut microbiota structure patterns were associated with sex or age of the host species, except for *H. gaumeri,* whose microbiota differed between juvenile and adult individuals (Table S4). Additionally, within species we found geographic structure for *H. gaumeri*, *O. phyllotis*, *P. yucatanicus* and *S. toltecus* (Table S4).

**Table 3:** Permanova test results based on weighted (Wunifrac) and unweighted (Unifrac) Unifrac distance dissimilarities and on Bray–Curtis index, quantifying the extent to which host species identity, geographic region, or their nested interaction explain beta diversity (species turnover)

	Estimator	df	*R*^2^	*F*	*p*-value	AICc	k	ω^2^
Host species	Bray–Curtis	5	0.2151	3.0158	0.001	−51.3265	6	0.114
	Wunifrac	5	0.1998	2.7472	0.001	−470.2741	6	0.125
	Unifrac	5	0.2613	3.8903	0.001	**−63.5331**	6	0.191
Geographic region	Bray–Curtis	2	0.1415	4.7792	0.001	**−52.9869**	3	0.110
	Wunifrac	2	0.1144	3.7459	0.001	**−471.2195**	3	0.082
	Unifrac	2	0.1642	5.6961	0.001	−63.1351	3	0.133
Host species/Geographic region	Bray–Curtis	12	0.3560	2.2113	0.001	−43.2027	13	0.192
	Wunifrac	12	0.4165	2.8551	0.001	−469.376	13	0.267
	Unifrac	12	0.4067	2.7424	0.001	−56.7221	13	0.255

AICc: Akaike information criterion corrected for small sample sizes; k: number of parameters in the model; ω^2^: Omega-squared values of effect size corrected for small sample size. Bold numbers indicate the best model for each estimator according to AICc

### Host Phylogenetic Signal

We assessed the influence of phylogeny on each diversity measure, considered as phenotypic traits, that characterize the microbiota of each rodent species; statistical independence between traits and phylogeny was determined with Blomberg’s *K* and Pagel’s λ. Results showed phylogenetic signals across all taxonomic levels for q0 (less for ASVs) and q1 (less for Class) (Table S5), namely that family richness and common taxonomic components of bacteria, respectively, have a phylogenetic component. Comparatively, dominant species (q2) did not have a phylogenetic signal. Additionally, a phylogenetic signal was found for beta dispersion (Fig. 2, Table S5), while beta diversity exhibited a positive association between phylogeny and microbiome dissimilarity, with average strength > 0.65 (Table S6). The phenetic trees showed that the tree topology changed among the microbial traits (q0, q1, q2) or taxonomic levels (Class, Order, Family, ASVs) (Fig. S6), mostly by *H. gaumeri* and *O. couesi* that consistently grouped with other rodent species. Particularly, *O. couesi* was associated with the Neotominae group for q0 (ASVs, Order) and q1 (ASVs), while it did for q1 (Family) and q2 (at all taxonomic levels) with its corresponding subfamily Sigmodontinae. Regarding *H. gaumeri*, it grouped with Neotominae for q0 and q1 (ASVs) and with Sigmodontinae for q0 (Order), q1 (Family), and q2 (Family).

## Discussion

Our findings indicate that both evolutionary (host species and phylogenetic signal) and ecological (geography and diet) drivers influence the gut microbiota structure and composition of the rodent community studied. Notably, results show shared but also distinct gut microbial diversity patterns associated with different habits of species. To our knowledge, our work is the first gut microbiota study of all the rodent species except *P. leucopus* [9].

### Gut Microbial Structure: Influence of Host Species Identity and Phylogeny

The microbial community structure in mammals is variable given that diet, geography, host genetics, environment, and phylogeny have been identified as key factors [1, 17, 23–26, 56]. However, it is uncertain how to separate the effect of these factors potentially nested in host phylogeny, and even less clear how they interact and shape microbial communities across diverse animal hosts [20]. Host phylogeny can constrain the interspecific composition, influencing both microbial alpha and beta diversity [19]. Concordantly, rodent host identity was identified as a factor structuring bacterial communities when beta phylodiversity was evaluated considering presence and absence data (unweighted Unifrac). Additionally, for all beta diversity statistics, the proportion of variance explained by the host species was ~ 30%. We acknowledge the limitation of the machine learning approach given the small sample size available; however, results showed high performance in classifying bacterial taxa by rodent species. Accordingly, our combined community structure findings support host species identity as a main factor determining the structure of bacterial communities in our study system.

Phylosymbiosis in sympatric mammals has been observed in African herbivores [21], rodents [20] and non-human primates [22], among others. According to [57], 15% of microbial structure and diversity in wild vertebrates can be explained by phylogeny, where phylosymbiosis is usually assessed using correlative methods. Remarkably, implementing the comparative method allowed us to model the evolution of alpha diversity and beta dispersion as phenotypic traits intrinsic to the host species, and identify which microbiota properties exhibit phylogenetic signals beyond species turnover. We acknowledge that a phylogeny with a low number of lineages, as is our case, can decrease the signal of the phylogenetic association [15]. Notwithstanding, we found a significant association between phylogeny and alpha microbiota richness (q0), common taxa (q1) and beta diversity (bacterial species replacement and beta dispersion), indicating that bacterial assembly dissimilarity increases with host phylogenetic distance. It is important to note that the strength (and significance) of the association between phylogeny and microbiota composition differed with the metric used to characterize the microbial communities.

Phylosymbiosis is often observed in recent host radiations [58], like in American Cricetidae species that experienced a rapid diversification, challenging phylogenetic reconstructions [59, 60]. Our results suggest that some levels of microbial diversity have a phylogenetic component, following the expected phylogenetic pattern; while others do not, like *H. gaumeri* and *O. couesi*. Ecological factors may counter or even disrupt the phylosymbiosis signal [61]. As shown by the phenograms, some *O. couesi* traits group with the Neotominae, the other omnivorous rodents; whereas in the PCoA and the phylogeny, it clusters with *S. toltecus*, both Sigmodontinae species. These results suggest that similar dietary niches in divergent rodent species are likely driving some microbial traits. In *H. gaumeri*, different traits were consistently associated with different host species, with no clear clustering even in the PCoA; such pattern suggests a strong environmental influence. Similar findings with Heteromyids exhibiting unique microbial dynamics have been reported [20]. Furthermore, the observed phylogenetic signal for beta dispersion is interesting since it reflects the variability of the microbiota of each host species, underpinning variation across geographic regions [2, 8, 12]; yet, ecological filtering likely constrains this variation, resulting in a microbial pool characteristic of each rodent species.

### Influence of Ecological Factors: Geography and Host Habits

Bacterial communities frequently exhibit geographic structuring patterns in wild animals due to ecological drift, *in-situ* diversification, or local selective pressures, for example in koalas (*Phascolarctos cinereus*), mice (*Mus musculus* and *Peromyscus maniculatus*), lemurs and muskoxen [24, 55, 62–64]. Our sampling size is limited, and not all species are found in the three geographic regions, a fact echoing the natural assembly, abundance, and distribution of these rodents in our study region, patterns that differed significantly across sampling sites. The hosts’ spatial distribution reduced the potential to determine the influence of geography on microbiota structure by rodent species; nonetheless, a significant signal was found for *P. yucatanicus*, *S. toltecus*, *O. phyllotis*, and *H. gaumeri*. Phylogeny-independent metrics like Bray–Curtis are most strongly influenced by dispersal and spatial patterns, while phylogeny-weighted metrics (Unifrac) reflect host life history [65]. Indeed, the high accuracy of the random forest algorithm to classify the differential bacterial taxa of the Mérida and Tizimín regions supports that host species, as well as geography, play an important role in determining the microbial communities.

This finding does not entirely agree with the idea that sympatric organisms often share the same microbiota because they share a microbial environmental pool (an inoculation source) [8, 12]. An environmental filtering process plays a complementary role in differentiating the species’ microbiota, regardless of the geographic region. Our results support a certain degree of structure by geography both in the global pool and by species, indicating that despite this microbial signature per rodent, there is some variation attributable to geography. Such patterns can be associated with the geographic distance among regions and their environmental differences (e.g., available food items, habitat modification), which can impose selective pressures and shape a contrasting assembly [61, 64, 66] of both the rodent hosts and their associated microbial community composition.

### Gut Microbiota Composition: Diet and Function

The dietary guild is typically associated with phylogeny, where closely related species often share similar dietary preferences and habits [25]. In fact, ecological factors such as diet and species habits explain 20–30% of bacterial diversity and structure in wild vertebrates, mostly in terms of selective pressures or environmental filtering [67, 68], including rodents [6]. Although our study was not focused on the effects of diet and our sampling is not suitable for testing it, the bacterial abundances associated with each species and their functional attributes further suggest that diet, jointly with the host phylogeny and geographic variation patterns, could be influencing gut microbiota differences among species. No detailed data on diet is known for most of our study species; *O. phyllotis* has been described to have a mainly folivorous-frugivorous diet, given its semi-arboreal habits, strongly associated with areas of dense vegetation [69]. *Sigmodon toltecus* diet consists of green plant material and fungi, and opportunistically of seeds and insects [70]. The diet of *P. yucatanicus* and *P. leucopus* consists of seeds, fruits, and arthropods [71, 72]. Heteromyids are characterized by cheek pouches where they store food, consuming mostly seeds and some fruits; *H. gaumeri* is known to also consume snails [73]. Lastly, *O. couesi,* which has semi-aquatic habits, eats seeds, fruits, herbs, crustaceans, and arthropods [74]. Herbivorous animals tend to have more diverse intestinal microbiomes compared to other omnivore or carnivore diets [6, 75], as the folivorous *O. phyllotis* which *s*tands out for its high bacterial richness.

We identified several bacterial families that can determine some of the main differences of the rodents’ gut microbiota, their abundances potentially associated with distinct habitats, diets, and metabolic functions [76]. Bifidobacteriaceae, which is abundant in *O. phyllotis,* specializes in degrading plant-derived glycans like glucans, fructans, xylans, pectins, cellulose and arabinoxylan. Members of Class Bacteroidia are characterized by the absence of cellulosome to degrade complex carbohydrates (e.g., pectins, cellulose, lignocellulose, xylans; [76, 77]), like the family Muribaculaceae that was also predominant in *O. phyllotis*. The CAZy enzymatic family present in Lachnospiraceae and Ruminoccocaceae also degrades plant glycans [76, 78]. Interestingly, the lignocellulose metabolism contributes to the serum composition of the metabolome, mainly short-chain fatty acids, providing substrate for other bacterial families and host metabolism [78]. These bacterial groups co-occur, with different abundances, in the species we studied, which have also been observed in the rodent *Rhizomys pruinosus* from Southeast Asia [77]. Rikenellaceae is common in rodents like *S. toltecus* while Ruminoccocaceae was the predominant family in *H. gaumeri*. According to [68], having one or the other indicates functional selection in herbivores. Finally, Desulfovibrionaceae was abundant in *O. couesi,* which are bacteria that metabolize hydrogen sulfide (H_2_S) by anaerobically reducing organic (taurine) or inorganic (sulfate and sulfite) sulfur gut components [79]. Particularly, taurine is derived in highest amounts from meat and seafood, which correlates with the diet reported for the semi-aquatic *O. couesi* [74]. Eggerthellaceae also showed differential enrichment in *O. couesi*; it can process isoflavones like daidzein and genistein, which are phytoestrogens, and multiple cinnamates that are produced by a variety of plants and fruits [80], likely associated with this species omnivorous diet. Interestingly, the *Peromyscus* species showed a high abundance of Rs-E47_termite_group, a bacterial family first described in termites, concordant with these omnivorous rodents including insects in their diets.

## Conclusion and Perspectives

The overall patterns of the six rodent species we found offer unique insights into the rodent community from three regions in the Yucatán peninsula, their gut microbiotas and their ecology. Our study focused explicitly on evaluating the influence of some evolutionary and ecological factors, attempting to understand the dynamics of sympatric rodent host communities and their microbiome patterns. Our findings show that both, ecology and evolution, influence these rodents gut microbial structure and composition, exhibiting shared and distinctive patterns. While most species showed phylosimbiosis, the environment played a significant role for species like *H. gaumeri*, and habits (omnivorous diet) showed a stronger signal for *O. couesi*. Rodents with more specialized habits like the semi-arboreal and folivorous *O. phyllotis* had higher bacterial diversity.

It is important to consider that some ecological traits like host environment, host diet, and host habits, as well as functional traits such as host morphology, body size and physiology are not necessarily independent of phylogeny. Such traits and their interactions could also contribute to the observed patterns, something we cannot fully discern with our data. Further work is needed to understand the mechanisms and processes shaping phylosymbiosis at different phylogenetic scales and across different microbiome traits. Studies need to continue evaluating the different levels of biological information contained in the microbiome and its association with the host. It is necessary to design and implement new analytical methods to evaluate phylosymbiosis not only based on species turnover along host divergence time but also considering specific features of the microbiome, such as diversity metrics and taxa abundance, to better determine the microbiome dynamics.

## Supplementary Information

Below is the link to the electronic supplementary material.

Supplementary file1 (PDF 1485 KB)

Supplementary file2 (XLSX 16 KB)

## Data Availability

The 16S raw sequence data are publicly available in NCBI under the Bioproject number PRNJNA1322533. Metadata (e.g., sampling locations, data per individual) and methods for analyses are available in the main text and in the Supplementary Information.
