# Supplementary material for: Evolutionary and Ecological Drivers of Gut Microbiota in Wild Rodent Species from the Yucatán Peninsula
Source: Microb Ecol. 2025 Sep 30;88:100. doi: 10.1007/s00248-025-02603-3 (PMC12484284; doi:10.1007/s00248-025-02603-3)
Supplement: Supplementary file 1 [file 248_2025_2603_MOESM1_ESM.pdf]

Evolutionary and ecological drivers of gut microbiota in wild rodent species from the Yucatán peninsula

Gabriela Borja-Martínez, Arit de León-Lorenzana, Alfredo Yanez, Giovani Hernández-Canchola, Luisa I. Falcón, Ella Vázquez-Domínguez

Supplementary information

Extended methods

Study site and sampling

Our study design targeted sampling in three geographic regions in the Yucatán peninsula, which are characterized by different land uses and habitat modification, classified as urban, rural and natural (Sánchez-Soto et al., 2024) respectively (Figure 1): i) Mérida, with 921,771 inhabitants, is the political, cultural and economic hub of the region. The leading causes of deforestation are urbanization and intensive poultry and pig farming; ii) Tizimín ('the tapir's place' in Maya) has 52,600 inhabitants and is the primary livestock producer in the peninsula; deforestation is mainly due to intensive non-rotating monocropping and livestock management. It encompasses a complex landscape matrix intermingled with rural, agricultural, and livestock zones as well as remnants of natural vegetation; iii) Calakmul ('two adjacent mounts' in Maya), is a natural and cultural World Heritage site recognized by UNESCO, and a Biosphere Reserve that comprises the largest tropical forest (dry, tall and medium-height subperennial rainforest) in Mexico (INEGI, 2020). Agriculture, livestock and illegal logging in the border zones are the main causes of land-use change, jointly with the development of tourism infrastructure.

The rodent community was live-trapped in three sites within each region, for two nights per site, using 120 Sherman traps (baited with a mixture of oats, vanilla extract and peanut butter) spaced 10 m apart along randomly set transects. Sampling was performed in the dry season in November 2021 (Mérida), March 2022 (Tizimín), and May 2022 (Calakmul). We replaced each trap with a clean one after rodent capture, always changing gloves and cleaning handling equipment to prevent cross-contamination. Each individual was identified to species, sexed and weighed, and standard morphological measures were taken (Supplementary Table S1); they were released at the sampling site assuring individual well-being. Our research was approved by the Comité de Ética en Investigación y Docencia ENES Mérida (OFICIO No. ENES/MID/CEID/010/2022), following the ethical standards of Universidad Nacional Autónoma de México, and with the corresponding collecting permit from Secretaría del Medio Ambiente y Recursos Naturales (Nº SGPA/DGVS/03783/21 and SPARN/DGVS/00459/22). Field sampling

and animal handling procedures strictly adhered to the American Society of Mammalogists guidelines for the use of wild mammal species (Sikes et al., 2016). We collected fresh fecal material in Eppendorf tubes that were preserved and sealed in liquid nitrogen.

DNA extraction and sequencing

DNA was extracted from fecal samples using the QIAamp PowerFecal Pro DNA Kit (Qiagen) following the manufacturer´s instructions. The quality of DNA was evaluated via agarose gels and with DNA absorbance ratio of 260/280 in Nanodrop. Total DNA per sample was quantified in Qubit, and all samples were diluted to 20 ng/ml. We amplified the V4 variable region of the 16S rDNA locus using primers 515F/806R (Caporaso et al., 2011) in triplicates per sample. PCR reactions (25 µL) consisted of 2.5 µL magnesium buffer, 2.0 µL dNTPs, 0.7 µL BSA, 0.125 µL taq, 0.5 µL of each primer, 17.7 µL water. PCR conditions included an initial denaturation at 94°C for 3 min, 35 cycles at 94°C for 45", 50°C for 60" and 72°C for 90", followed by a final elongation at 72°C for 10 min. Successful amplification was verified with agarose gel electrophoresis. PCR products were pooled and purified with SPRI beads (NimaGen, NDL). The lab workflow was performed in a laminar flow cabinet with negative controls of all the buffers used for the DNA extraction and amplification. Negative controls did not generate any PCR product thus they were not sequenced. The libraries were sequenced on Illumina MiSeq, using the Illumina 16S metagenomic sequencing library Prep Kit, in the Yale Center for Genome Analysis (YCGA).

Microbial abundance, alpha and beta diversity

To characterize the gut microbiota per rodent species, we assessed abundance differences of microbial taxa among host species with a linear discriminant effect size analysis (lefse) using microbiomeMarker v.1.4 (Cao et al., 2022). Lefse calculates abundance differences by converting read counts to a percentage; next, it uses Kruskal-Wallis sum-rank test to identify differential abundance between classes and the effect size (>2) with linear discriminant analysis (Nearing et al., 2022). We further identified the core gut microbiota and the shared taxa at the family level among host species based on their relative abundances and on their 95% and 75% prevalence, with microbiome v.1.2 (Lahti & Shetty, 2017). We also evaluated the unique and shared taxa at the genus level, characterizing each host species with microViz (Barnett et al., 2021) and with the DrawVenn tool available online (https://bioinformatics.psb.ugent.be/webtools/Venn/).

Phylogenetic relationships

To evaluate phylogenetic signals, we first obtained a phylogenetic tree for the rodent species studied. We downloaded cytochrome *b* sequences available in GenBank for our study species: *H. gaumeri* (Accession number: GU646999), *P. leucopus* (DQ973104), *O. phyllotis* (DQ179814), *S. toltecus* (EU073182), *O. fulvescens* (EU258547), and *O. couesi* (DQ185386). No sequence was available for *P. yucatanicus*; thus we amplified, sequenced, and aligned one sample (GenBank number: PQ742163), following Hernández-Canchola et al. (2021). We selected the best model and partition scheme, divided by codon positions, using AICc with PartitionFinder2 (Lanfear et al., 2016). We built an ultrametric tree with BEAST 1.10.4 (Suchard et al., 2018), performing two independent runs, 10 million generations and sampling every 1000 iterations. We evaluated the convergence of runs with Tracer 1.7 (Rambaut et al., 2018) by examining trace plots and sample sizes >200; we combined results with 20% burn-in with LogCombiner and built a maximum credibility tree with TreeAnnotator.

References

Barnett, D. J., Arts, I. C., & Penders, J. (2021). microViz: an R package for microbiome data visualization and statistics. *Journal of Open Source Software*, *6*(63), 3201. https://doi.org/10.21105/joss.03201

Cao, Y., Dong, Q., Wang, D., Zhang, P., Liu, Y., & Niu, C. (2022). microbiomeMarker: An R/Bioconductor package for microbiome marker identification and visualization. *Bioinformatics*, *38*(16), 4027-4029. https://doi.org/10.1093/bioinformatics/btac438

Caporaso, J. G., Lauber, C. L., Walters, W. A., Berg-Lyons, D., Lozupone, C. A., Turnbaugh, P. J., Fierer, N., & Knight, R. (2011). Global patterns of 16S rRNA diversity at a depth of millions of sequences per sample. *Proceedings of the National Academy of Sciences*, *108*, 4516-4522. https://doi.org/10.1073/pnas.1000080107

Hernández-Canchola, G., León-Paniagua, L., & Esselstyn, J. A. (2021). Mitochondrial DNA indicates paraphyletic relationships of disjunct populations in the *Neotoma mexicana* species group. *Therya*, *12*(3), 411-421. https://doi.org/10.12933/therya-21-1082

INEGI. (2020). Uso de suelo y vegetación. https://www.inegi.org.mx/temas/usosuelo/ (Accessed 17 May 2024)

Lahti, L., & Shetty, S. (2017). microbiome R package. Bioconductor. https://doi.org/10.18129/B9.bioc.microbiome

Lanfear, R., Frandsen, P. B., Wright, A. M., Senfeld, T., Calcott, B. (2017). PartitionFinder 2: new methods for selecting partitioned models of evolution for molecular and morphological phylogenetic analyses. *Molecular Biology and Evolution*, *34*, 772-773, https://doi.org/10.1093/molbev/msw260

Nearing, J. T., Douglas, G. M., Hayes, M. G., MacDonald, J., Desai, D. K., Allward, N., Jones, C. M. A., Wright, R. J., Dhanani, A. S., Comeau, A. M., & Langille, M. G. I. (2022). Microbiome differential abundance methods produce different results across 38 datasets. *Nature Communications*, *13*(1), 342. https://doi.org/10.1038/s41467-022-28034-z

Rambaut, A., Drummond, J., Xie, D., Baele, G., Suchard, M. A. (2018). Posterior summarization in Bayesian phylogenetics using Tracer 1.7. *Systematic Biology*, *67*, 901-904. https://doi.org/10.1093/sysbio/syy032

Sánchez-Soto, Ma. F., Gaona, O., Vigueras-Galván, A. L., Suzán, G., Falcón, L. I., & Vázquez-Domínguez, E. (2024). Prevalence and transmission of the most relevant zoonotic and vector-borne pathogens in the Yucatan peninsula: A review. *PLOS Neglected Tropical Diseases*, *18*(7), e0012286. https://doi.org/10.1371/journal.pntd.0012286

Suchard, M. A., Lemey, P., Baele, G., Ayres, D. L., Drummond, A.J., Rambaut, A. (2018). Bayesian phylogenetic and phylodynamic data integration using BEAST 1.10. *Virus Evolution*, *4*(1), vey016. doi: 10.1093/ve/vey016

Figures S1 — S6

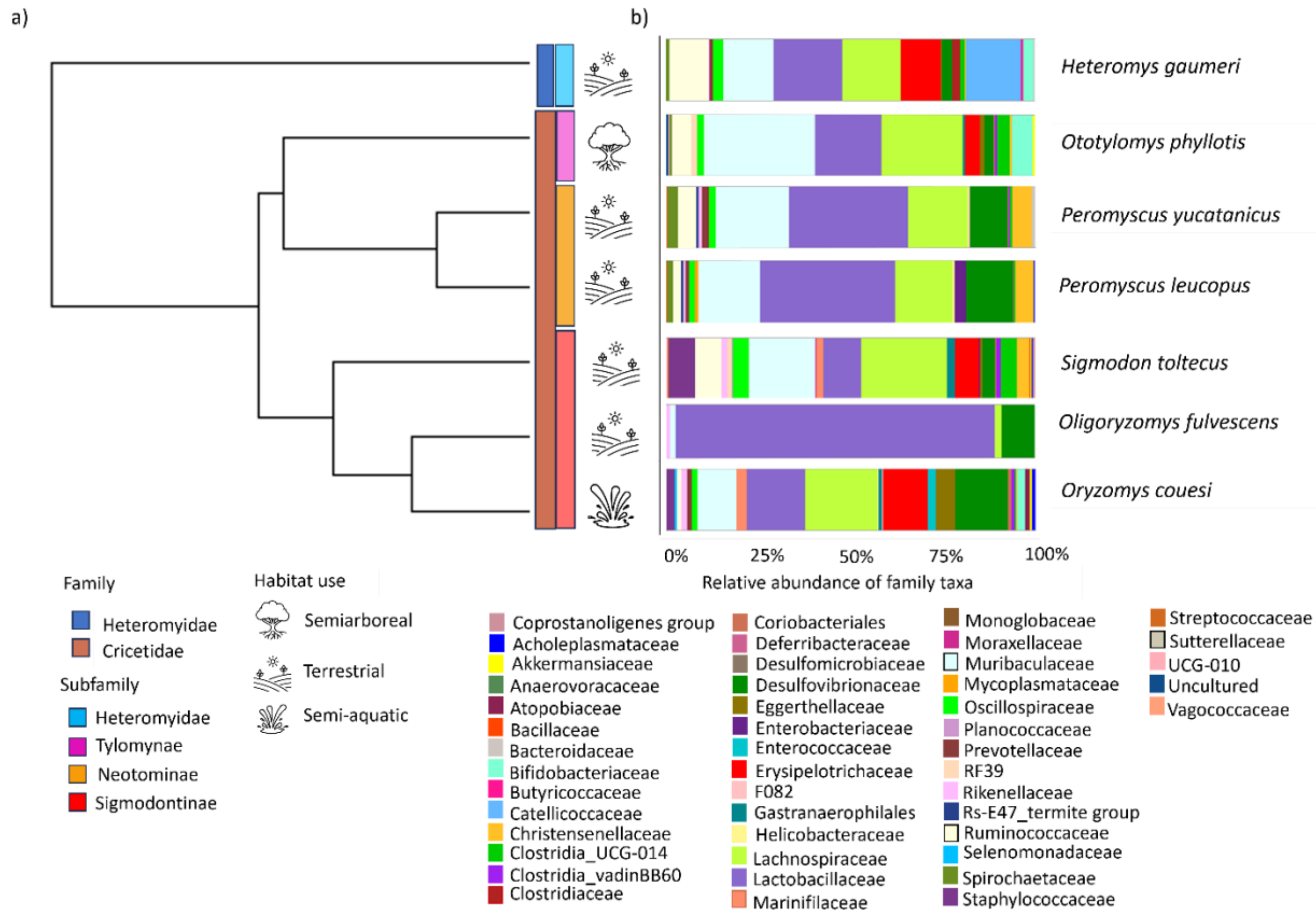

Figure S1. a) Phylogeny of the rodent species studied based on cytochrome *b* sequences. Taxonomic classification (Family and Subfamily) and habitat use (semiarboreal, terrestrial, semi-aquatic) of each species are indicated. **b**) Relative abundance of bacteria families (higher than 0.01; colored bars) of the gut microbiota of each host rodent species.

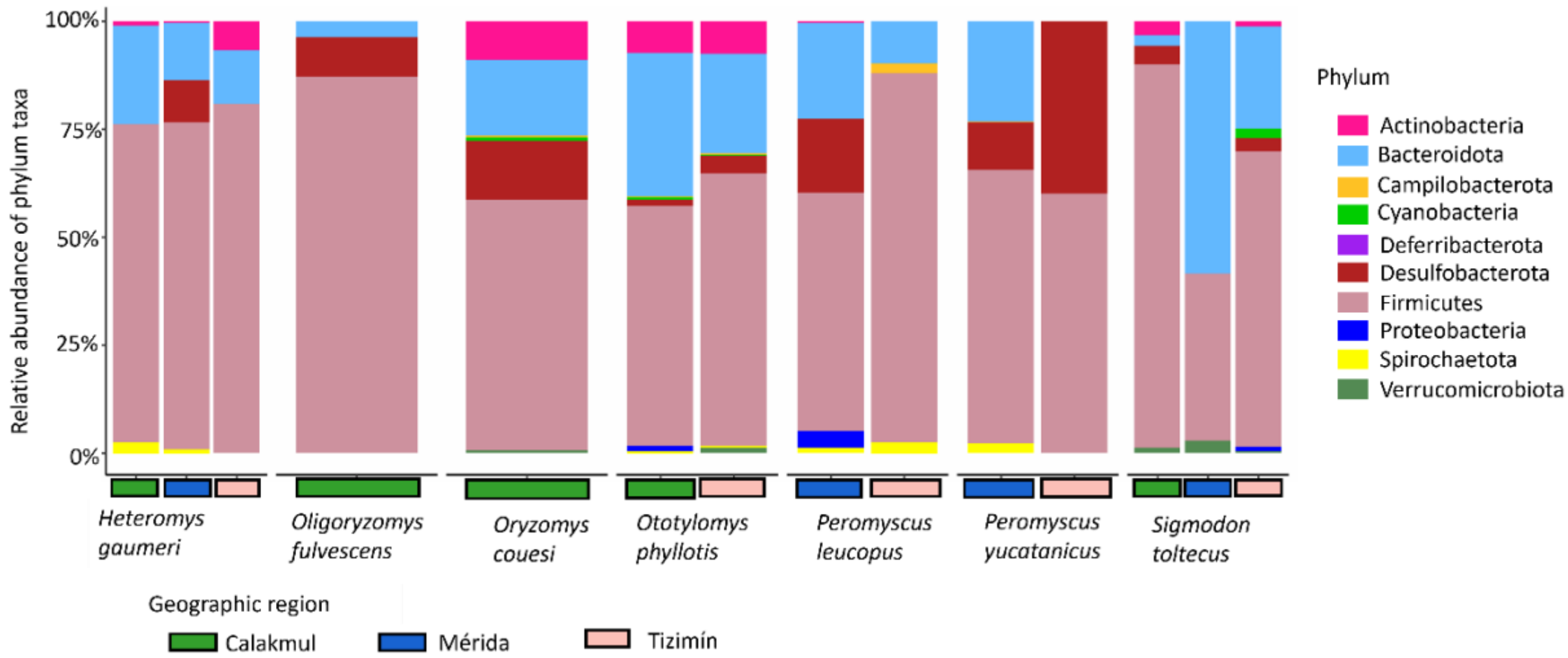

Figure S2. Relative abundance (higher than 0.01; colored bars) of the gut bacteria at the Phylum level for each host species. Colored bars in the bottom indicate the geographic distribution of the species, showing Calakmul in green, Mérida in blue and Tizimín in pink.

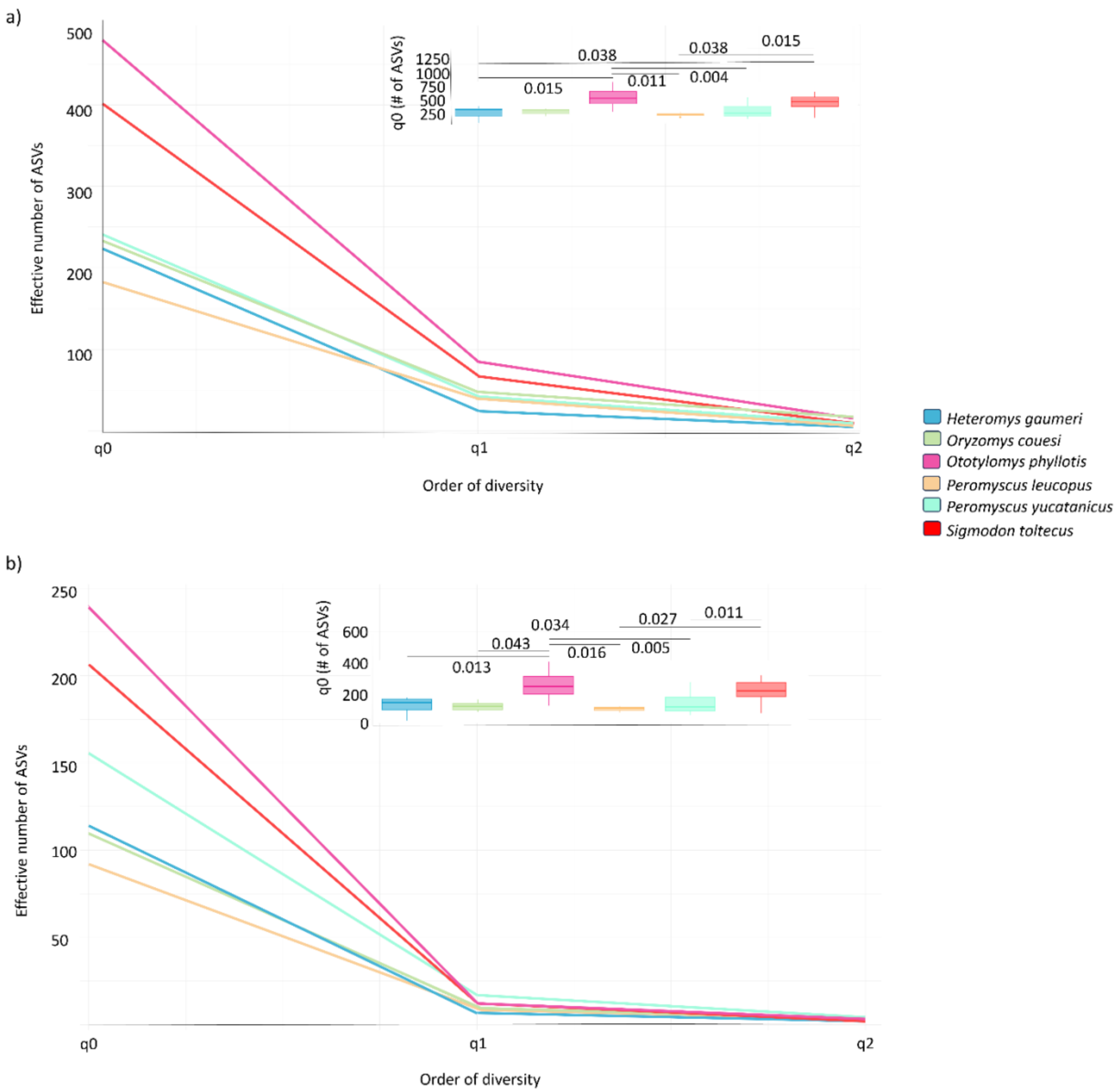

Figure S3. Diversity (effective number of ASVs) profiles of the microbial community by host species. Alpha diversity and phylodiversity among host species are shown in the top (**a**) and bottom (**b**) panels, respectively. Significant comparisons among species are indicated (top right insert in each graph), where q0: species richness, q1: number of common species, and q2: number of dominant species.

a)

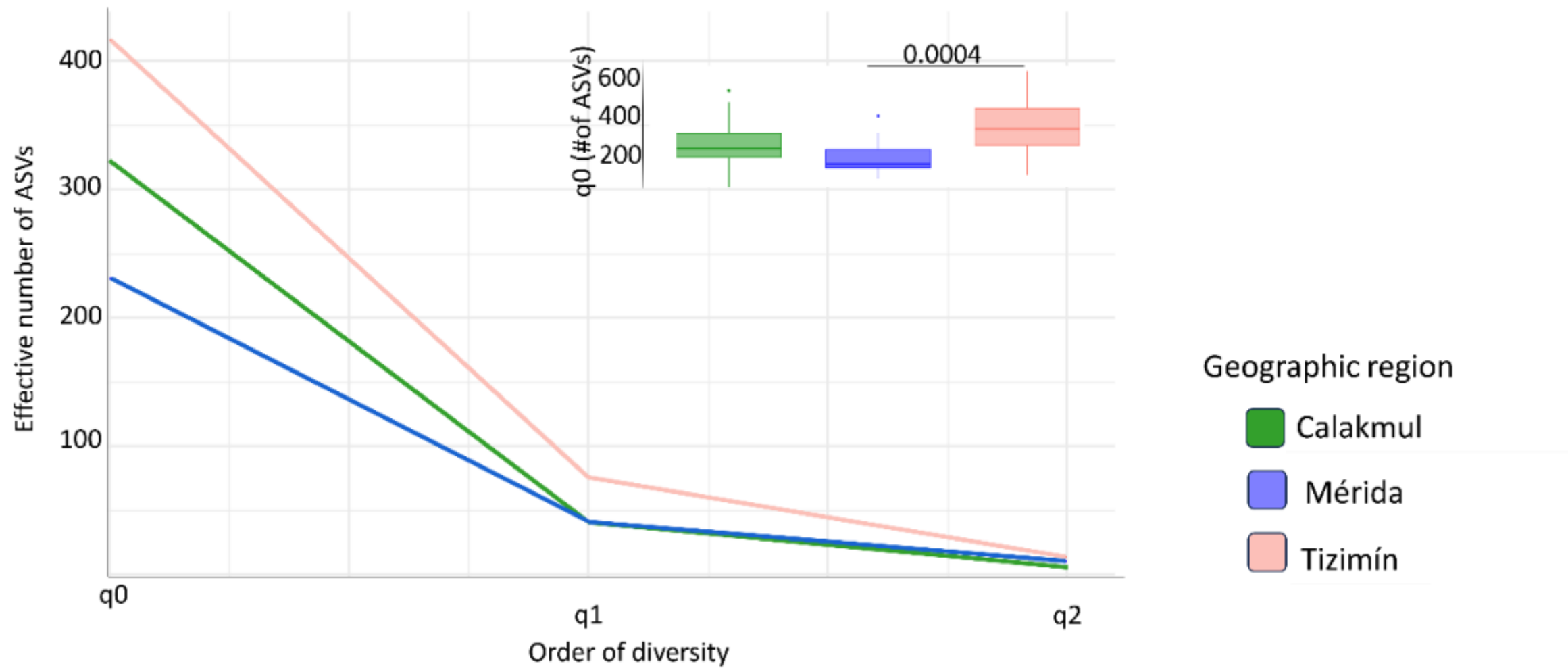

b)

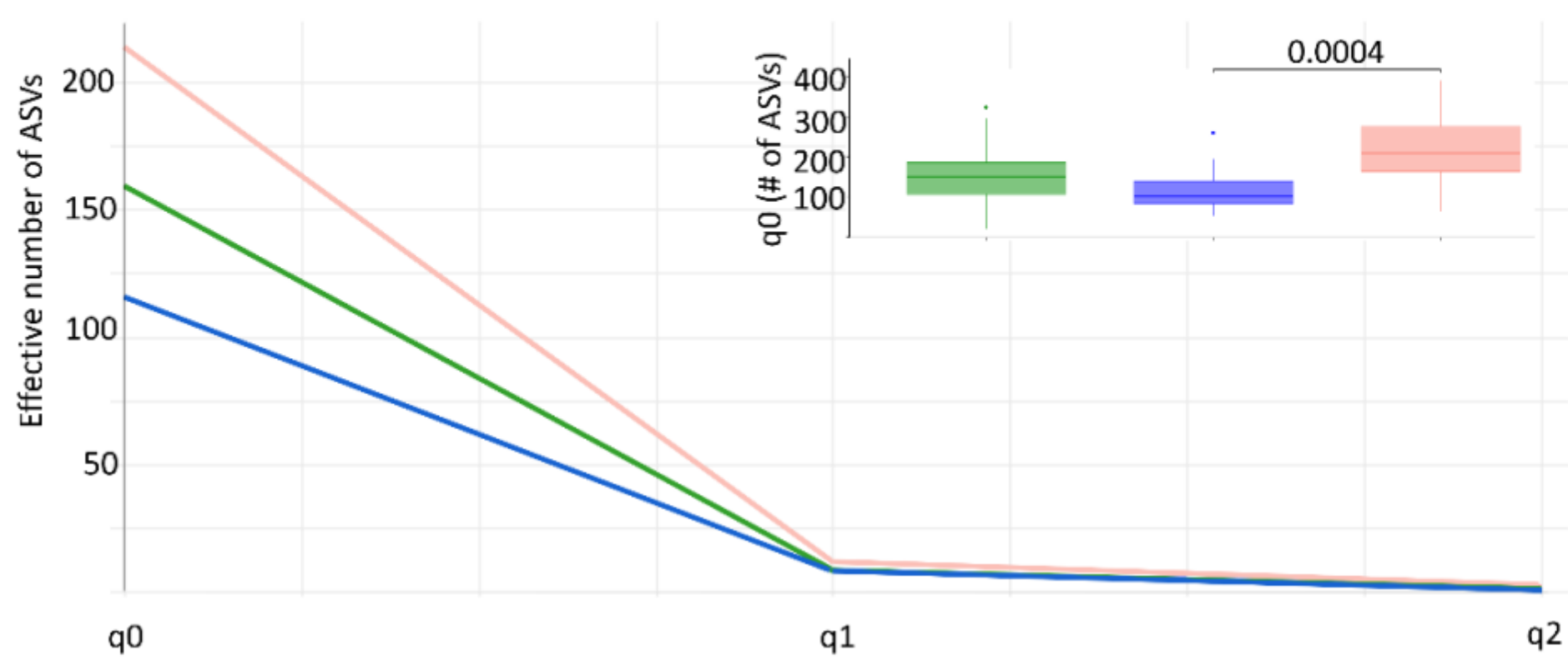

Figure S4. Diversity (effective number of ASVs) profiles of the microbial community by geographic region. Alpha diversity and phylodiversity among regions are shown in the top (**a**) and bottom (**b**) panels, respectively. Significant comparisons between regions at q0 level are indicated (top right insert in each graph). q0: species richness, q1: number of common species, q2: number of dominant species.

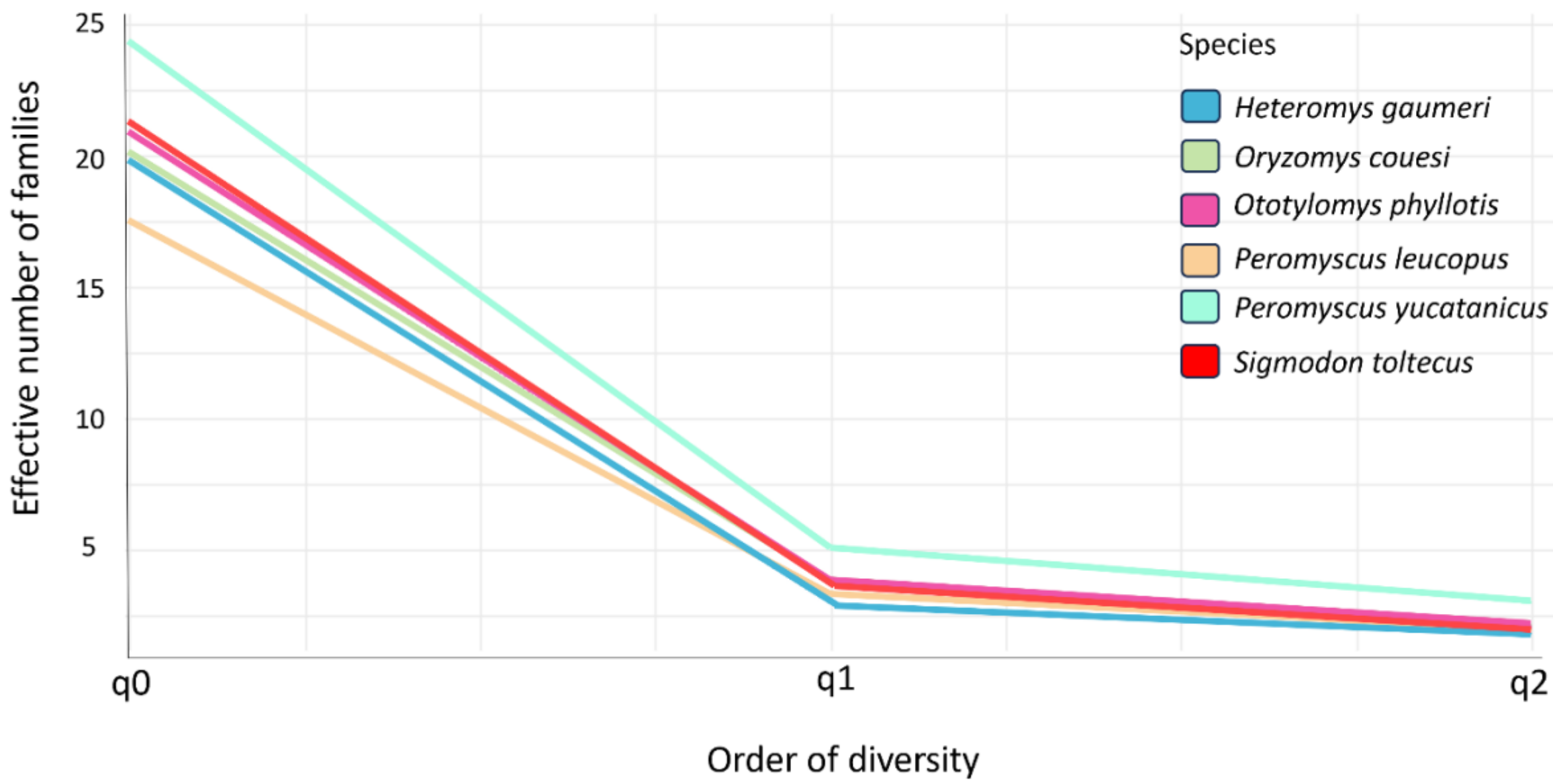

Figure S5. Phylodiversity (effective number of families) profiles of the microbial community by host species. Alfa phylodiversity among host species is shown, where q0: species richness, q1: number of common species, and q2: number of dominant species.

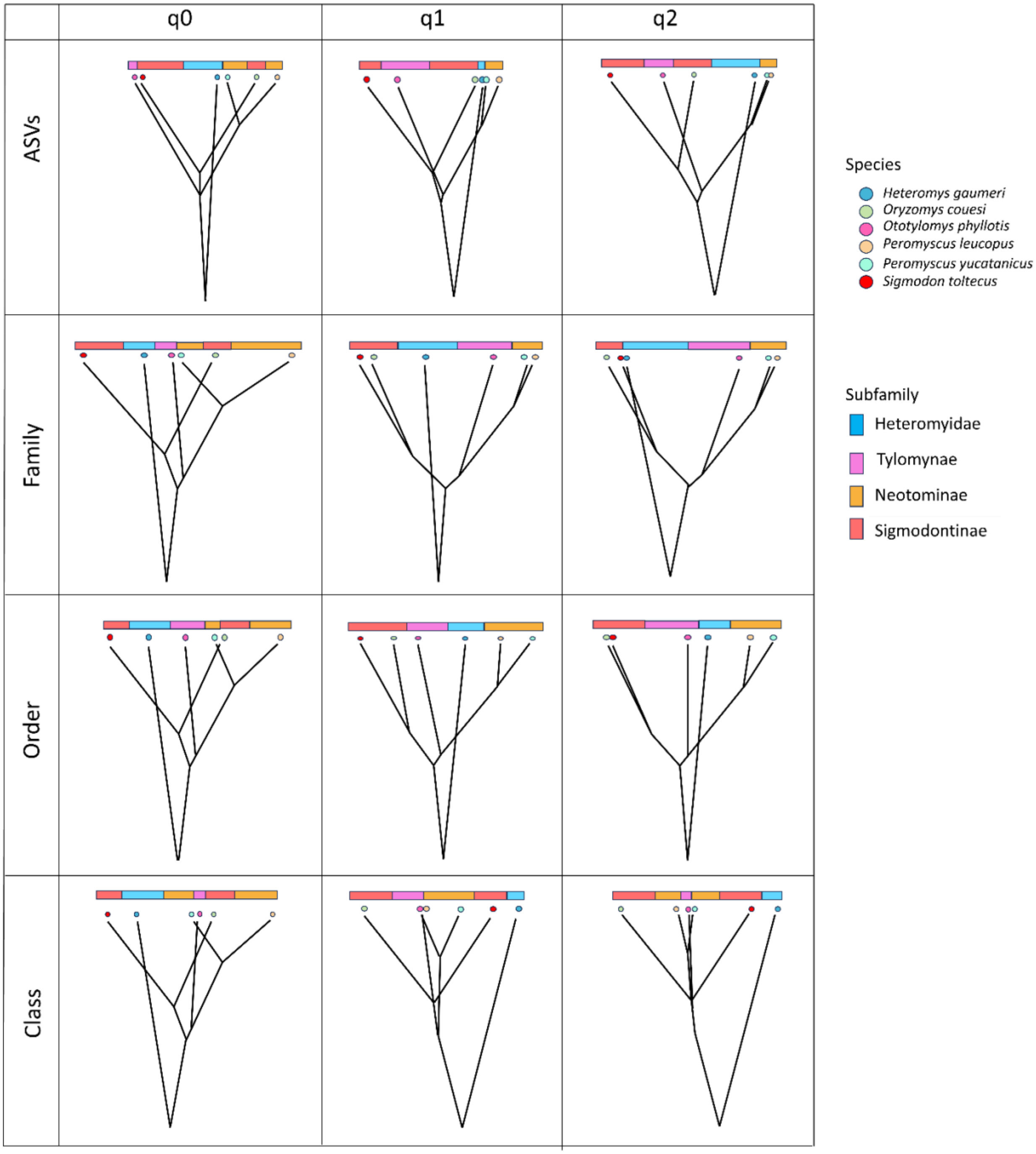

Figure S6. Phenetic tree for the rodent species studied. microbial traits. The panels show how some species change position along the tree branches according to the microbial trait (q0, q1, q2) and the taxonomic level (Class, Order, Family, ASVs).

Tables S1 — S6

Table S1. Sampling information and morphological data per individual of the rodent community from three sampling regions in the Yucatán peninsula, Mexico (**provided as an independent excel file**).

Table S2. Shared and unique gut microbial taxa among the six rodent host species studied. Gaumer's spiny pocket mouse (*Heteromys gaumeri*), Coues's rice rat (*Oryzomys couesi*), big-eared climbing rat (*Ototylomys phyllotis*), white-footed mouse (*Peromyscus leucopus*), Yucatan deer mice (*Peromyscus yucatanicus*) and Toltec cotton rat (*Sigmodon toltecus*).

**Host Species**	**Phylum**	**Taxonomic assignment at genus level**
H. gaumeri<br>*O. couesi*<br>*O. phyllotis*<br>*P. leucopus*<br>*P. yucatanicus*<br>*S. toltecus*	Firmicutes	[Eubacterium]_brachy_group
		[Eubacterium]_coprostanoligenes_group
		[Eubacterium]_siraeum_group
		[Eubacterium]_ventriosum_group
		[Eubacterium]_xylanophilum_group
		A2
		Anaeroplasma
		Butyricicoccus
		Clostridia_UCG-014
		Clostridia_vadinBB60_group
		Colidextribacter
		Enterococcus
		GCA-900066575
		Lachnoclostridium
		Lachnospiraceae_NK4A136_group
		Lachnospiraceae_UCG-001
		Lachnospiraceae_UCG-006
		Lactobacillus_amylolyticus
		Lactobacillus uncultured
		Lactococcus
		Monoglobus
		Mycoplasma
		NK4A214_group
		Oscillibacter
		RF39
		Roseburia
		Ruminococcus
		Staphylococcus

		UCG-003
		UCG-005
		UCG-010
	Actinobacteriota	Enterorhabdus
		Uncultured
	Bacteroidota	Alistipes
		Bacteroides
		Odoribacter
	Desulfobacterota	Desulfovibrio
	Cyanobacteria	Gastranaerophilales
	Campilobacterora	Campylobacter
		Helicobacter_winghamensis
		Helicobacter
	Crenarchaeota	Candidatus_Nitrososphaera
	Elusimicrobiota	Elusimicrobium
	Proteobacteria	Escherichia_coli
		Uncultured
H. gaumeri<br>*O. couesi*<br>*O. phyllotis*<br>*P. yucatanicus*<br>*S. toltecus*	Firmicutes	Cellulosilyticum
		Peptococcus
		Ruminiclostridium
		UCG-009
H. gaumeri<br>*O. phyllotis*<br>*P. leucopus*<br>*P. yucatanicus*<br>*S. toltecus*	Firmicutes	ASF356
		Blautia
		Catellicoccus
		Coprococcus
		Ruminococcaceae
		Ruminococcus
	Bacteroidota	Muribaculum
	Euryarchaeota	Methanobrevibacter
	Desulfobacterota	Desulfomicrobium
	Proteobacteria	Enterobacter
H. gaumeri<br>*O. couesi*<br>*P. leucopus*<br>*P. yucatanicus*<br>*S. toltecus*	Firmicutes	Bacillus
	Actinobacteriota	Rothia
	Bacteroidota	Parabacteroides
O. couesi<br>*O. phyllotis*<br>*P. leucopus*<br>*P. yucatanicus*<br>*S. toltecus*	Firmicutes	Lachnospiraceae_FCS020_group
	Verrucomicrobiota	Akkermansia

H. gaumeri<br>*O. couesi*<br>*O. phyllotis*<br>*P. leucopus*<br>*P. yucatanicus*	Firmicutes	Lactobacillus_reuteri
	Deferribacterota	Mucispirillum
	Euryarchaeota	Methanosphaera
	Proteobacteria	Oxalobacter
	Patescibacteria	Candidatus_Saccharimonas
	Spirochaetota	Termite_Treponema_cluster
	Myxococcota	bacteriap25
H. gaumeri<br>*O. couesi*<br>*O. phyllotis*<br>*S. toltecus*	Firmicutes	Allobaculum
		Anaerofustis
		Dubosiella
	Actinobacteriota	Adlercreutzia
	Proteobacteria	uncultured_Alphaproteobacteria
H. gaumeri<br>*O. phyllotis*<br>*P. yucatanicus*<br>*S. toltecus*	Firmicutes	Family_XIII_UCG-001
		Monoglobus
		Ruminococcus_torques_group
		Streptococcus
		Tuzzerella
	Proteobacteria	Uncultured
H. gaumeri<br>*O. couesi*<br>*P. yucatanicus*<br>*S. toltecus*	Firmicutes	Eubacterium_nodatum_group
H. gaumeri<br>*P. leucopus*<br><br>*P. yucatanicus*<br>*S. toltecus*	Proteobacteria	Nitrosomonas
O. phyllotis<br>*P. leucopus*<br>*P. yucatanicus*<br>*S. toltecus*	Firmicutes	Clostridium_methylpentosum_group
		Fournierella<br><br>Heteromys Oryzomis Oto Yucatanicus
H. gaumeri<br>*O. couesi*<br>*O. phyllotis*<br>*P. yucatanicus*	Firmicutes	Ruminococcus _uncultured_rumen
H. gaumeri<br>*O. couesi*<br>*P. leucopus*<br>*P. yucatanicus*	Bacteroidota	Prevotellaceae_UCG-001
		Rikenellaceae_RC9_gut_group
		Rs-E47_termite_group
H. gaumeri	Actinobacteriota	Bifidobacterium

O. couesi<br>*O. phyllotis*<br>*P. leucopus*		
O. couesi<br>*O. phyllotis*<br>*P. yucatanicus*<br>*S. toltecus*	Firmicutes	UCG-005
	Crenarchaeota	Nitrososphaeraceae
	Proteobacteria	Morganella_morganii
O. couesi<br>*P. leucopus*<br>*P. yucatanicus*<br>*S. toltecus*	Firmicutes	Lachnospiraceae_UCG-001
		Lachnospiraceae_UCG-006
		Roseburia
		Lachnoclostridium
		Ruminococcus
H. gaumeri<br>*O. phyllotis*<br>*S. toltecus*	Firmicutes	Christensenellaceae_R-7_group
		Defluviitaleaceae_UCG-011
		Lachnospiraceae_NK4B4_group
		Marvinbryantia
		Tyzzerella
	Bacteroidota	Rikenella
H. gaumeri<br>*P. leucopus*<br>*S. toltecus*	Firmicutes	Streptococcus
O. couesi<br>*O. phyllotis*<br>*S. toltecus*	Firmicutes	Anaerovorax
	Bacteroidota	Butyricimonas
		ML635J-40_aquatic_group
H. gaumeri<br>*P. yucatanicus*<br>*S. toltecus*	Firmicutes	[Eubacterium]_ventriosum_group
	Cyanobacteria	Scytonema_VB-61278
H. gaumeri<br>*P. leucopus*<br>*P. yucatanicus*	Bacteroidota	Prevotella
		Alloprevotella
		Prevotellaceae_UCG-003
	Verrucomicrobiota	Candidatus_Xiphinematobacter
H. gaumeri<br>*O. phyllotis*<br>*P. yucatanicus*	Firmicutes	Mycoplasma
	Proteobacteria	Pantoea
O. phyllotis<br>*P. yucatanicus*<br>*S. toltecus*	Firmicutes	Acetatifactor
		Eubacterium_fissicatena_group
		Eubacterium_ruminantium_group
		Fournierella
		Roseburia
		UBA1819
O. phyllotis<br>*P. leucopus*<br>*S. toltecus*	Firmicutes	Bacteroides_pectinophilus_group

O. phyllotis *P. leucopus* *P. yucatanicus*	Verrucomicrobiota	Coraliomargarita
		uncultured
O. couesi *P. yucatanicus* *S. toltecus*	Firmicutes	Lysinibacillus
O. couesi *P. leucopus* *P. yucatanicus*	Bacteroidota	F082
	Verrucomicrobiota	Victivallis
P. leucopus *P. yucatanicus* *S. toltecus*	Actinobacteriota	Corynebacterium
		Kocuria_palustris
	Proteobacteria	Acinetobacter
	Verrucomicrobiota	Chlamydia
H. gaumeri *O. couesi* *O. phyllotis*	Firmicutes	Faecalibaculum
		Holdemanella
		Ileibacterium
	Actinobacteriota	Olsenella
	Proteobacteria	Parasutterella
H. gaumeri *S. toltecus*	Firmicutes	Eubacterium_nodatum_group
		Clostridium_sensu_stricto_1
		Lachnospira
		Negativibacillus
	Actinobacteriota	Bifidobacterium
		Bifidobacterium_animalis
		Corynebacterium
		Mycobacterium
H. gaumeri *O. phyllotis*	Firmicutes	Frisingicoccus
H. gaumeri *P. yucatanicus*	Firmicutes	NK4A214_group
	Actinobacteriota	Streptomyces_sp.
O. phyllotis *S. toltecus*	Firmicutes	Agathobacter
		Butyricicoccus
		Eubacterium_ruminantium_group
		Family_XIII_AD3011_group
		Izemoplasmatales
		Lachnoclostridium
		UBA1819
	Actinobacteriota	Collinsella
	Desulfobacterota	Bilophila
		Desulfovermiculus
O. couesi *S. toltecus*	Firmicutes	Psychrobacillus
		Solibacillus
	Desulfobacterota	Uncultured
	Proteobacteria	Parasutterella
O. couesi	Actinobacteriota	OPB41

O. phyllotis		
P. yucatanicus *S. toltecus*	Firmicutes	Acetatifactor
		Hathewaya
		Sporosarcina_pasteurii
		uncultured
		Ureaplasma
	Actinobacteriota	Uncultured
O. phyllotis *P. yucatanicus*	Firmicutes	Candidatus_Arthromitus
		Oscillibacter
		Peptococcus
	Desulfobacterota	Desulfovibrio_piger
	Spirochaeota	Treponema
P. leucopus *P. yucatanicus*	Proteobacteria	Kosakonia
H. gaumeri	Firmicutes	Anaerotruncus
		Anaerovibrio
		Candidatus_Arthromitus
		Candidatus_Soleaferrea
		Coprococcus
		Faecalibacterium
		Harryflintia
		Lachnospiraceae_UCG-008
		Lachnospiraceae_UCG-009
		UCG-005
		uncultured
		V9D2013_group
	Actinobacteriota	67-14
		DNF00809
		Nonomuraea
		Pseudonocardia
		Raoultibacter
		Rubrobacter
	Bacteroidota	Parabacteroides
		Prevotellaceae_NK3B31_group
	Proteobacteria	Citrobacter
		Pasteurellaceae_bacterium
		Rodentibacter
O. couesi	Firmicutes	Cohnella
		Halolactibacillus
		Lactobacillus uncultured
		Paenibacillus
		Veillonellaceae_UCG-001
	Bacteroidota	Prevotellaceae_UCG-004
		Bacteroidales_RF16_group
	Proteobacteria	Aureimonas

		Providencia_sp.
O. phyllotis	Firmicutes	Butyrivibrio
		Acetitomaculum
		Agathobacter
		Anaerostignum
		Anaerovorax
		Blautia
		Family_XIII_UCG-001
		Hydrogenoanaerobacterium
		Intestinimonas
		Lachnoclostridium scindens
		Lachnospiraceae_ND3007_group
		Lachnospiraceae_NK3A20_group
		UCG-003
		UCG-007
		V9D2013_group
	Bacteroidota	p-251-o5
	Proteobacteria	Herbaspirillum
	Spirochaeota	GWE2-31-10
P. leucopus	Proteobacteria	Pseudomonas
P. yucatanicus	Firmicutes	Anaerocolumna
		Carnobacterium
		Enterococcus_faecium
		Lachnospiraceae_AC2044_group
		Lachnospiraceae_NK4A136_group
		Lactobacillus_brevis
		Lactococcus_lactis
		Oribacterium
		Pelosinus
		Robinsoniella
		Selenomonas_ruminantium
		Vagococcus
		Weissella
	Proteobacteria	Ensifer_adhaerens
		Methylobacterium-Methylorubrum
		Serratia_sp
S. toltecus	Firmicutes	Aerococcus
		Aerosphaera
		Alkalibacterium
		Ammoniphilus
		CAG-352
		Christensenellaceae_R-7_group
		Clostridium_novyi

		Dielma
		Domibacillus
		Eisenbergiella
		Epulopiscium
		Eubacterium_siraeum_group
		Eubacterium_ventriosum_group
		Family_XIII_AD3011_group
		Fictibacillus
		Kurthia
		Lachnospiraceae_FCS020_group
		Lachnospiraceae_NK3A20_group
		Oceanobacillus
		Paludicola
		Paenibacillus
		Pediococcus_pentosaceus
		Peptoniphilus
		RF39
		Romboutsia
		Salinicoccus
		Sporosarcina
		Streptococcus_agalactiae
		Streptococcus_dysgalactiae
		Streptococcus_pluranimalium
		Symbiobacterium
		Syntrophomonas
		Terribacillus_goriensis
		Turicibacter
		UCG-004
		Weissella_viridescens
		Weissella_paramesenteroides
		Weissella_jogaejeotgali
	Actinobacteriota	Actinomyces
		Actinoplanes
		Brevibacterium
		Cellulomonas
		Cellulosimicrobium
		Corynebacterium_diphtheriae
		Curtobacterium
		Dietzia
		Glutamicibacter
		Microbacterium

		Nocardioides
		Patulibacter
		Rhodococcus_sp.
		Solirubrobacter
		Streptomyces
		Trueperella
	Bacteroidota	Butyricimonas
		Porphyromonas
	Proteobacteria	Acinetobacter
		Allorhizobium-Neorhizobium-Pararhizobium-Rhizobium
		Devosia
		Hyphomicrobium
		Methylobacterium_hispanicum
		Methylobacterium-Methylorubrum
		Microvirga
		Ochrobactrum
		Pseudomonas
		Sphingomonas

Table S3. Confusion matrix obtained by Support vector machine (SVM) and random forest (RF). The accuracy of the classification model to assess differences between the gut microbiota by host species and by geographic region is indicated.

	**Precision**	**Recall**	**F1 score**	**Support**	**Global accuracy**		
Heteromys gaumeri	0	0	0	1	0.75	**Support vector machine**	**Host species**
Ototylomys phyllotys	0.95	0.75	0.80	4.2			
Peromyscus complex	0.64	1.0	0.77	5.2			
Sigmodon toltecus	0.86	0.70	0.74	5.6			
Heteromys gaumeri	0.37	0.37	0.33	1	0.94	**Random forest**	
Ototylomys phyllotys	0.90	1.0	0.95	4.2			
Peromyscus complex	1.0	1.0	1.0	5.2			
Sigmodon toltecus	0.96	0.73	0.93	5.6			
Calakmul	0	0	0	1.8	0.76	**Support vector machine**	**Geographic region**
Mérida	0.82	0.90	0.85	6			
Tizimín	0.75	0.84	0.78	8.2			
Calakmul	0.46	0.46	0.42	1.8	0.83	**Random forest**	
Mérida	0.97	0.96	0.96	6			
Tizimín	0.84	0.81	0.82	8.2			

Table S4. Permanova test results based on weighted (Wunifrac) and unweighted (Unifrac) distance dissimilarities and on Bray-Curtis index, quantifying the extent to which geographic region, sex, and age explained beta diversity (species turnover) by rodent species. ω^2: Omega-squared values of effect size corrected for small sample size. Bold numbers indicate a significant microbial structure.

**Species**	**Factor**	**Estimator**	**df**	R^2	ω^2	*F*	***p*-value**
Heteromys gaumeri	Geography	Bray-Curtis	2	0.50954	0.23544	2.0778	**0.013**
		Unifrac	2	0.46165	0.16965	1.7151	**0.009**
		Wunifrac	2	0.38407	0.065955	1.2471	0.348
	Sex	Bray-Curtis	1	0.18879	0.022837	1.1636	0.359
		Unifrac	1	0.16131	-0.0055096	0.96164	0.453
		Wunifrac	1	0.17262	0.0061311	1.0432	0.4
	Age	Bray-Curtis	1	0.27836	0.11713	1.9287	0.062
		Unifrac	1	0.22432	0.059893	1.446	**0.045**
		Wunifrac	1	0.13019	-0.037289	0.74836	0.629
Oryzomys couesi	Sex	Bray-Curtis	1	0.48984	-0.013453	0.96018	0.6667
		Unifrac	1	0.46792	-0.041879	0.87941	0.6667
		Wunifrac	1	0.57571	0.10632	1.3569	0.3333
	Age	Bray-Curtis	1	0.48984	-0.013453	0.96018	0.6667
		Unifrac	1	0.46792	-0.041879	0.87941	0.6667
		Wunifrac	1	0.57571	0.10632	1.3569	0.3333
Ototylomys phyllotis	Geography	Bray-Curtis	1	0.12283	0.032285	1.4003	**0.013**
		Unifrac	1	0.1081	0.017357	1.212	**0.046**
		Wunifrac	1	0.12341	0.032866	1.4078	0.055
	Sex	Bray-Curtis	1	0.0924	0.001504	1.0181	0.454
		Unifrac	1	0.09443	0.0035468	1.0427	0.355
		Wunifrac	1	0.10107	0.010251	1.1243	0.3
	Age	Bray-Curtis	1	0.06741	-0.023641	0.72286	0.968
		Unifrac	1	0.08709	-0.0038469	0.95401	0.503
		Wunifrac	1	0.09318	0.0022921	1.0276	0.441
Peromyscus leucopus	Geography	Bray-Curtis	1	0.44215	0.12763	1.5852	0.25
		Unifrac	1	0.38055	0.054075	1.2287	0.5
		Wunifrac	1	0.33384	0.0005673	1.0023	0.5
	Sex	Bray-Curtis	1	0.38885	0.063784	1.2725	0.5
		Unifrac	1	0.27128	-0.068222	0.74454	0.75
		Wunifrac	1	0.64699	0.3999	3.6656	0.25
	Age	Bray-Curtis	1	0.2413	-0.10008	0.6361	1
		Unifrac	1	0.42655	0.10867	1.4877	0.25
		Wunifrac	1	0.17723	-0.1659	0.43083	0.75
Peromyscus	Geography	Bray-Curtis	2	0.14148	0.11025	4.7792	**0.001**

yucatanicus		Unifrac	2	0.16417	0.13343	5.6961	**0.001**
		Wunifrac	2	0.11439	0.082592	3.7459	**0.001**
	Sex	Bray-Curtis	0.063334	0.00084835	1.0144	0.392	0.392
		Unifrac	1	0.05116	-0.011372	0.80885	0.945
		Wunifrac	1	0.06156	-0.00093998	0.98404	0.479
	Age	Bray-Curtis	1	0.08347	0.021084	1.3661	0.073
		Unifrac	1	0.06059	-0.0019148	0.96751	0.562
		Wunifrac	1	0.06405	0.001552	1.0264	0.406
Sigmodon toltecus	Geography	Bray-Curtis	2	0.14926	0.026136	1.2281	**0.019**
		Unifrac	2	0.15207	0.029166	1.2554	**0.033**
		Wunifrac	2	0.29372	0.18356	2.911	**0.005**
	Sex	Bray-Curtis	1	0.0674	0.0049235	1.0841	0.214
		Unifrac	1	0.06367	0.0011791	1.0201	0.381
		Wunifrac	1	0.06179	-0.00071622	0.98783	0.385
	Age	Bray-Curtis	1	0.06405	0.0015534	1.0265	0.42
		Unifrac	1	0.05881	-0.0036994	0.93734	0.692
		Wunifrac	1	0.0603	-0.0022112	0.96249	0.424

Table S5. Trait similarity among host species. Statistical independence of traits among host species, considering their phylogenetic relationships based on our phylogenetic tree (see Fig. 1 main text). The phenotypic traits evaluated were gut microbiota diversity metrics alpha and phylodiversity (q0, q1, q2) and beta dispersion (for definitions see Methods in main text). Blomberg´s *K* and Pagel´s λ statistic tests comparing if trait similarity at different taxonomic levels (ASVs, Family, Order, Class) was greater than expected following a random-walk model along the branches of the phylogeny. Index values close to zero (< 1) indicate phylogenetic independence; p-value if the index value is different from zero.

**Microbiota diversity**		**Pagel´s λ**		**Bloomberg´s K**	
		**Index**	**p-value**	**Index**	**p-value**
Beta dispersion	Wunifrac	1.0678	0.206	1.4523	**0.004**
	Bray-Curtis	1.1833	0.147	1.3729	**0.008**
ASV_q0	Alfa diversity	5.08E-05	1	0.7643	0.382
	Phylodiversity	5.08E-05	1	0.7681	0.372
ASV_q1	Alfa diversity	1.2450	0.407	0.7489	0.381
	Phylodiversity	1.2483	**0.025**	0.8735	0.294
ASV_q2	Alfa diversity	5.08E-05	1	0.6479	0.497
	Phylodiversity	1.1362	0.233	1.3655	0.087
Family_q0	Alfa diversity	1.2069	0.157	1.1952	**0.044**
	Phylodiversity	1.2390	0.079	1.1447	0.107
Family_q1	Alfa diversity	1.0084	0.362	1.1626	**0.039**
	Phylodiversity	1.1484	0.162	1.3329	**0.031**
Family_q2	Alfa diversity	0.5045	0.873	0.9231	0.214
	Phylodiversity	1.0268	0.234	1.3817	0.076
Order_q0	Alfa diversity	1.2168	0.129	1.2445	**0.047**
	Phylodiversity	1.2320	0.108	1.1797	0.067
Order_q1	Alfa diversity	1.2483	**0.008**	1.1078	0.088
	Phylodiversity	1.2447	**0.047**	1.1607	0.073
Order_q2	Alfa diversity	1.1438	0.230	1.2393	0.053
	Phylodiversity	1.2191	0.108	1.3765	**0.045**
Class_q0	Alfa diversity	1.0560	0.233	1.4626	**0.009**
	Phylodiversity	5.39E-01	1	0.6522	0.551
Class_q1	Alfa diversity	1.2393	0.176	1.1854	0.091
	Phylodiversity	5.39E-01	1	0.7852	0.389
Class_q2	Alfa diversity	5.39E-01	1	0.8856	0.221
	Phylodiversity	5.39E-01	1	0.8843	0.262

Table S6. Beta diversity and phylogenetic signal. Mantel correlation tests between gut microbiota beta diversity (Unifrac and Bray-Curtis) at different taxonomic levels (ASVs, Genus, Family, Order, Class) and phylogenetic distance.

Microbiota beta-diversity		Mantel test	
		R2	p-value
ASV	Bray-Curtis	0.6406	**0.0111**
	Unifrac	0.7668	0.0930
Genus	Bray-Curtis	0.6508	**0.0166**
	Unifrac	0.7175	**0.0277**
Family	Bray-Curtis	0.5257	**0.0194**
	Unifrac	0.6855	**0.0347**
Order	Bray-Curtis	0.5373	**0.0236**
	Unifrac	0.6878	**0.0333**
Class	Bray-Curtis	0.2058	0.2347
	Unifrac	0.3463	0.2597
